# Global change effects on biogeochemical mercury cycling

**DOI:** 10.1007/s13280-023-01855-y

**Published:** 2023-03-29

**Authors:** Jeroen E. Sonke, Hélène Angot, Yanxu Zhang, Alexandre Poulain, Erik Björn, Amina Schartup

**Affiliations:** 1 Géosciences Environnement Toulouse, CNRS/IRD, Université Paul Sabatier Toulouse 3, 14 ave Edouard Belin, 31400 Toulouse, France; 2 Univ. Grenoble Alpes, CNRS, INRAE, IRD, Grenoble INP, IGE, 1025 rue de la piscine, 38000 Grenoble, France; 3 School of Atmospheric Sciences, Nanjing University, 163 Xianlin Road, Nanjing, 210023 Jiangsu China; 4 Department of Biology, University of Ottawa, Ottawa, ON K1N6N5 Canada; 5 Department of Chemistry, Umeå University, 90187 Umeå, Sweden; 6 Geosciences Research Division, Scripps Institution of Oceanography, University of California San Diego, 9500 Gilman Drive, La Jolla, CA 92093 USA

**Keywords:** Climate change, Environment, Exposure, Fish consumption, Minamata Convention, Toxicity

## Abstract

Past and present anthropogenic mercury (Hg) release to ecosystems causes neurotoxicity and cardiovascular disease in humans with an estimated economic cost of $117 billion USD annually. Humans are primarily exposed to Hg via the consumption of contaminated freshwater and marine fish. The UNEP Minamata Convention on Hg aims to curb Hg release to the environment and is accompanied by global Hg monitoring efforts to track its success. The biogeochemical Hg cycle is a complex cascade of release, dispersal, transformation and bio-uptake processes that link Hg sources to Hg exposure. Global change interacts with the Hg cycle by impacting the physical, biogeochemical and ecological factors that control these processes. In this review we examine how global change such as biome shifts, deforestation, permafrost thaw or ocean stratification will alter Hg cycling and exposure. Based on past declines in Hg release and environmental levels, we expect that future policy impacts should be distinguishable from global change effects at the regional and global scales.

**Supplementary Information:**

The online version contains supplementary material available at 10.1007/s13280-023-01855-y.

## Introduction

Human activities have released mercury to air, land and water at large scale since the sixteenth century, when cinnabar (HgS) was transformed to liquid Hg for use in silver and gold mining (UNEP 2018). Subsequent industrialization in the nineteenth and twentieth centuries has seen continued releases of Hg from silver and gold mining, in addition to losses from Hg mining and production, technological applications of Hg, chemical manufacturing, and since the 1960s from coal burning and metallurgy (Streets et al. 2017). International awareness of Hg toxicity has resulted from the dramatic methyl-Hg (MeHg) poisoning incidents in Minamata, Japan, and in Iraq in the 1960s and 1970s (McAlpine and Araki 1958). Subsequent research has quantified the global extent of Hg pollution, partly due to its atmospheric dispersal in the long-lived gaseous Hg^0^ form, but also the widespread microbial methylation of Hg in anoxic environments, and Hg biomagnification in aquatic food webs (EPA 1997). Parallel epidemiology studies in the 1980s and 1990s suggested neurodevelopmental toxicity by MeHg exposure from seafood consumption even at low doses (NRC/NAS 2000), and more recently cardiovascular disease in adults has been associated with MeHg exposure (Roman et al. 2011). The global nature of Hg pollution, its large impact in terms of human health and associated economic costs (Zhang et al. 2021a), have propelled international action in form of the 2013 UNEP Minamata Convention on Hg, which came into force in 2017. The Minamata Convention aims to curb Hg release to the environment and is accompanied by global Hg monitoring efforts to track its success.

However, a major challenge facing the Hg research and policy communities will be to understand if future Hg trends reflect successful policy or are, instead, caused by global change effects on Hg cycling. For the purpose of this synthesis, we define global change as the combined effects of changes in Hg emissions, climate, land-use and land cover, biodiversity and food web interactions, amongst others. The large scale biogeochemical changes associated with global change are not fully understood or quantified but are expected to influence Hg levels in biota and human exposures (Krabbenhoft and Sunderland 2013). In their exhaustive review Obrist et al. discuss the anticipated impacts from human and natural perturbations on global Hg cycling, concluding that ‘legacy’ Hg emissions will continue to affect the global cycle for decades to centuries (Obrist et al. 2018). Legacy Hg refers to the large amounts of Hg released since the sixteenth century, that are sequestered at contaminated sites or have moved downstream into rivers, reservoirs, wetlands and coastal sediments (Streets et al. 2019a). Re-emission of legacy Hg from soils and oceans is today two times larger than new anthropogenic emissions to air (Amos et al. 2013; UNEP 2018). The various contaminated Hg pools may remobilize or stay sequestered depending on changes in land use, land cover, flooding, fires, ocean dynamics, etc. In this synthesis, we focus on recent progress made on the topic of biogeochemical Hg cycling and global change and provide recommendations on research and monitoring needs. Global change also impacts organism health by modifying diet and nutrition, the microbiome, infectious disease, co-exposure to other toxicants and genetics. These topics are not covered here and we refer the reader to Eagles-Smith et al. for an extensive analysis (Eagles-Smith et al. 2018).

## Global biogeochemical mercury cycling

### Modern-day global Hg budget

**Fig. 1:**
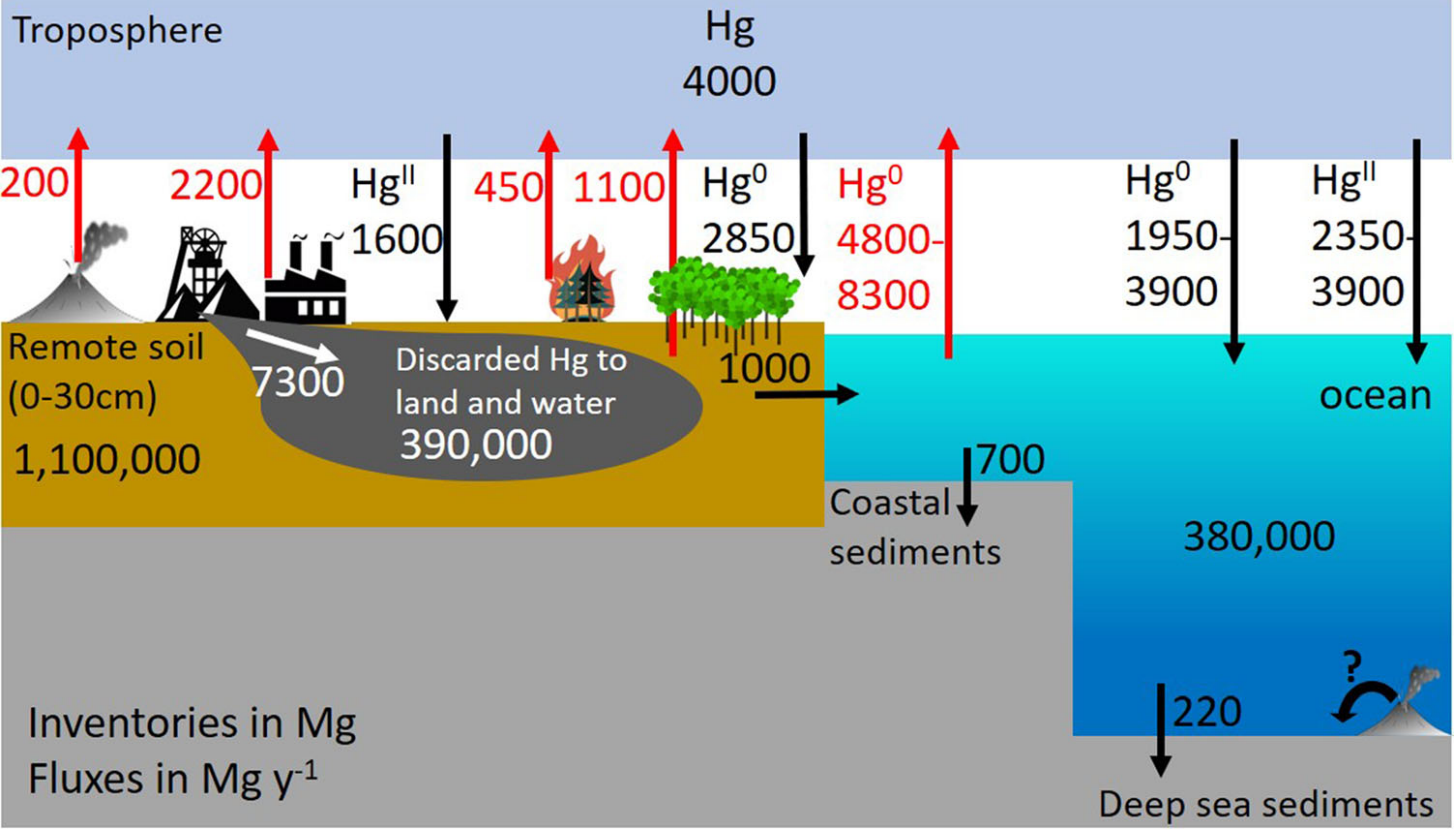
Modern day global Earth surface Hg cycling budget. Important differences from previous budgets are the revised volcanic emissions (Li et al. 2020), river transport and coastal sedimentation (Liu et al. 2021), deep sea sedimentation (Hayes et al. 2021), and enhanced vegetation and soil Hg^0^ uptake of 2850 Mg year^−1^. Important uncertainties remain for atmosphere–ocean exchange of Hg. All-time anthropogenic Hg release to land and water is estimated to be 1 070 000 Mg, of which 390 000 Mg are still sequestered at contaminated sites and supplied annually by 7300 Mg year^−1^ of new Hg releases (Streets et al. 2017). Terrestrial remote soil and discarded Hg pools both drive modern river Hg flux and re-emission to air. Major reservoir Hg inventories were also updated: atmosphere (Shah et al. 2021), soil (Lim et al. 2020) and ocean (Zhang et al. 2020). All other fluxes are from Shah et al. (2021)

**Fig. 2:**
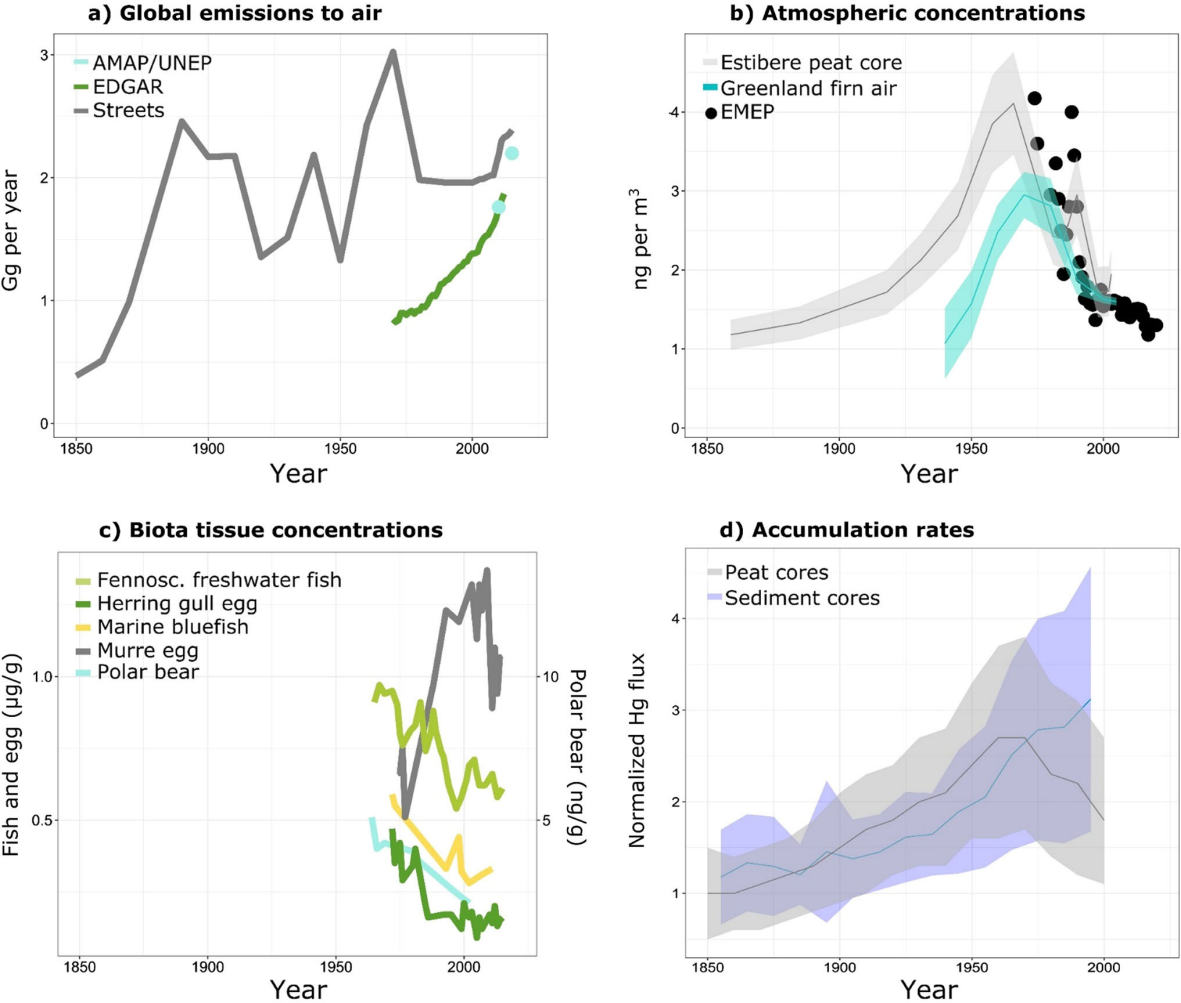
**a** Historical primary global anthropogenic Hg emissions since 1850 according to different emission inventories: AMAP/UNEP (UNEP 2018), EDGARv4.tox2 (Muntean et al. 2018), and Streets (Streets et al. 2019a, 2019b) inventories are shown in blue, green, and grey, respectively. **b** Atmospheric concentrations from EMEP monitoring stations (annual medians; in black) and reconstructed atmospheric concentrations from the Estibere peat core (Enrico et al. 2017; in grey) and Greenland firn air (Fain et al. 2009; in blue). The shaded region represents one standard deviation confidence interval. **c** Standardized biota tissue Hg concentrations trends for > 38 year long time-series from Fennoscandian freshwater fish (μg g^−1^ wet weight; Braaten et al. 2017), US east coast marine bluefish (μg g^−1^ wet weight; Cross et al. 2015), Svalbard polar bear dental tissue (ng g^−1^ dry weight; Aubail et al. 2012), and murre egg Hg (μg g^−1^ dw) from North-Canada (Braune et al. 2016) and herring gull eggs from Lake Superior (Blukacz-Richards et al. 2017). **d** Hg accumulation rates (normalized Hg flux to pre-industrial levels) in peat cores (*n* = 30; Enrico 2015) and remote lake sediment cores (*n* = 68; Zhang et al. 2014). The shaded region represents the standard deviation

**Fig. 3:**
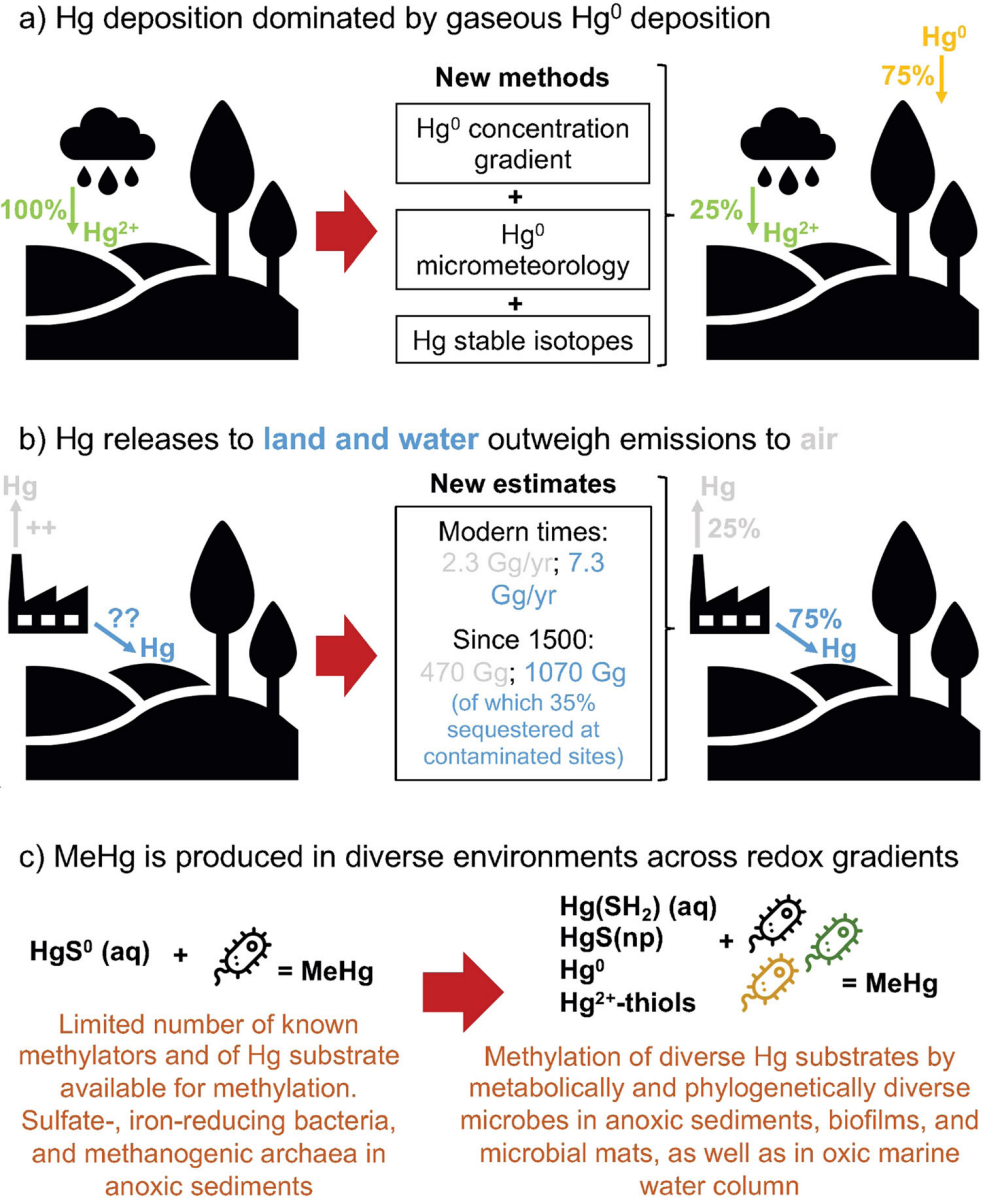
Paradigm shifts relevant to global change effects on Hg cycling. **a** The wealth of monitoring data since the 1990s on Hg wet deposition has fueled a paradigm where wet and dry Hg^II^ deposition are the main routes of terrestrial and marine ecosystem loading (UNEP 2003, 2018). Deposition of gaseous Hg^0^ (and minor Hg^II^) forms is far more difficult to quantify. Only since 2005 have new methods, including Hg^0^ concentration gradient methods (Obrist et al. 2017), Hg^0^ micrometeorology (Skov et al. 2006; Osterwalder et al. 2016), and Hg stable isotopes provided a different picture (Demers et al. 2013; Enrico et al. 2016). These methods now confirm, across all biomes, that direct foliar and soil Hg^0^ uptake ‘deposits’ far more atmospheric Hg to soils (75%), and to lakes and coasts, than Hg^II^ wet and dry deposition alone (25%) (Zheng et al. 2016; Obrist et al. 2018). Important global Hg^0^ deposition to land and oceans implies different climate change sensitivity where factors such as (de-)forestation need to be considered (Feinberg et al. 2022). **b** Hg release to air, land and water. Much emphasis has been put on the volatility of Hg, in terms of its emissions and atmospheric cycling and dispersal. Only since the 2010s have robust estimates of global Hg release to land and freshwater ecosystems been made (Horowitz et al. 2014; Streets et al. 2017), and indicate that these have been much larger both in the past (1070 Gg to land and water since 1510, vs. 470 Gg to air) and in modern times (7.3 Gg year^−1^ to land and water in 2010, vs. 2.3 Gg year^−1^ to air) than Hg emissions to air. Historically, release to land and water was dominated by Hg mining and production, Ag mining, and chemical manufacturing (Streets et al. 2017). It is estimated that on the order of 390 Gg of legacy Hg release to land and water is sequestered at contaminated sites (Fig. 1; Streets et al. 2019a), while the remaining 680 Gg is dispersed in the soil–river–wetland–coastal sediment continuum. Extreme events associated with climate change, or land-use change and deforestation may therefore mobilize substantial amounts of sequestered legacy Hg from contaminated sites into aquatic ecosystems. **c** Microbial Hg methylation studies identified sulfate and iron reducing bacteria, and methanogenic archaea as key methylating species, leading to a paradigm where MeHg was mainly produced in anoxic sediments with dissolved Hg^II^-sulfide species as substrate. The discovery of the hgcA and hgcB genes (Parks et al. 2013) and recent methodological developments in metagenomics are shaping a new paradigm, characteristic of a far larger diversity of methylating microbes across the full redox spectrum, and methylating a diverse set of Hg substrates (Regnell and Watras 2019)

A solid understanding of the Hg cycle and its underlying mechanistic complexity is critical to assess its response to global change. Our understanding of the biogeochemical Hg cycle has improved over the past four decades, including knowledge on natural and anthropogenic Hg sources, Hg dispersal, Hg biogeochemistry and Hg–food web dynamics. Figure 1 describes the state of knowledge on the global inorganic Hg cycle, based on observations and modeling. Natural Hg release includes aerial volcanic emissions of 200 ± 60 Mg year^−1^ and crustal degassing of 135 ± 40 Mg year^−1^ (Li et al. 2020). The magnitude of submarine hydrothermal Hg release is uncertain (Fitzgerald and Lamborg 2004), and is assumed here to transfer to local deep sea sediments without impacting marine surface waters. Global anthropogenic emissions to air in 2015 were around 2200 Mg year^−1^ (Figs. 1, 2a). New anthropogenic emissions to air therefore outweigh natural emissions by a factor of 7. Anthropogenic Hg emissions to air are overwhelmed, in turn, by the 3 times larger new anthropogenic Hg releases to land and water (7300 Mg year^−1^; Figs. 1, 3 Streets et al. 2017). Hg emissions from land, including natural crustal degassing, are estimated to be 1100 Mg year^−1^ in models (Horowitz et al. 2017; Shah et al. 2021), which is within the large uncertainty of observations of 600 Mg year^−1^ (− 500 to 1350, 37.5th and 62.5th percentiles; Agnan et al. 2016), with an additional terrestrial emission of 450 Mg year^−1^ from wildfires (Kumar et al. 2018; Shah et al. 2021).

Observed atmospheric Hg concentrations are relatively stable from one year to the next, indicating that the sum of estimated global Hg emissions must be approximately balanced by atmospheric Hg deposition. Global Hg deposition in Fig. 1 includes estimates of 1600 Mg year^−1^ total Hg^II^ (wet and dry Hg^II^) deposition to land. Based on recent novel methods and data, we revise vegetation and soil uptake of Hg^0^ up from 1200 (Horowitz et al. 2017; Shah et al. 2021) to 2850 ± 500 Mg year^−1^ (Obrist et al. 2021; Zhou and Obrist 2021; Feinberg et al. 2022). Hg^II^ deposition over oceans and Hg^0^ ocean uptake and evasion are difficult to measure and hence to model. Recent marine Hg stable isotope observations were able to constrain the relative contributions of atmospheric Hg^0^ and Hg^II^ deposition to the oceans as approximately 1:1 (Jiskra et al. 2021). Jiskra et al. argue that in previous budgets either Hg^II^ deposition to the ocean was overestimated, or the magnitude of Hg^0^ gas exchange was underestimated. A new study employing a coupled atmosphere–land–ocean model investigated these two scenarios and concluded on the likeliness of higher Hg^0^ gas exchange fluxes (Zhang et al. 2023). The budget Fig. 1 depicts this by the large uncertainty range of atmosphere–ocean Hg exchange. Terrestrial Hg is also transferred to coastal oceans by river inputs, which were recently estimated to amount to 1000 Mg year^−1^ (Liu et al. 2021). The global Hg budget in Fig. 1 suggests that global atmospheric Hg deposition is dominated by gaseous Hg^0^ uptake pathways (55%), in particular due to the large vegetation Hg^0^ uptake flux (Fig. 3). Below we will discuss what repercussions this has for global change effects on global Hg cycling and exposure.

After 500 years of anthropogenic Hg release to the environment, most Hg in surface Earth reservoirs that are critical to Hg dispersal and exposure, i.e., surface soils, atmosphere, wetlands, lakes, coastal zone and surface ocean, is of anthropogenic origin. It is estimated that 1070 Gg of Hg has been released to land and water since 1500, and 470 Gg of Hg to air (Fig. 3 Streets et al. 2019a). Global top soils (0–30 cm) are estimated to contain around 1100 Gg of Hg, oceans 380 Gg, and the atmosphere 4 Gg (Fig. 1). Soils and oceans are temporary reservoirs for Hg, which exits its short-term Earth surface cycle by deposition to coastal sediments (700 Mg year^−1^ Liu et al. 2021) and to deep sea sediments (220 Mg year^−1^ Hayes et al. 2021). Even today, new natural and anthropogenic releases to Earth surface reservoirs exceed burial fluxes, indicating net accumulation of Hg in Earth surface reservoirs. Natural Hg concentrations, stocks and fluxes can still be found in deep soils and sediments, deep groundwaters and possibly the deep ocean.

### Past trends in Hg release, loading and exposure

The extent to which humans have polluted environmental reservoirs over centuries or millennia is often expressed in terms of enrichment factors. Natural archives such as lake and peat sediments and ice cores have been used to estimate Hg enrichment in deposition, and by inference in the atmosphere. Natural archive studies generally agree that since the start of the industrial revolution around 1850 ce Hg deposition to remote locations has increased by a factor of 3 to 5 (Zhang et al. 2014; UNEP 2018; Li et al. 2020). This is reflected in Fig. 2a with a sharp increase in global emissions to air after 1850. Longer ^14^C dated sediment and peat cores generally show an earlier increase in Hg deposition in the northern hemisphere, but not the southern hemisphere, around 1500 ce (Li et al. 2020). All-time Hg enrichment factors in deposition, comparing modern to pre-1500 ce Hg deposition rates, reached their maximum of 16-fold at the height of twentieth century Hg release and are currently around tenfold. A tenfold enrichment in modern (1990–2010) Hg deposition broadly agrees with the sevenfold larger global anthropogenic Hg emission flux of 2200 Mg year^−1^ compared to the natural Hg emission flux of 340 Mg year^−1^ (Fig. 1).

Reconstructed atmospheric Hg concentrations from firn air (Fain et al. 2009) and peat archives (Enrico et al. 2017) suggest atmospheric Hg to have peaked at 3–4 ng m^−3^ in the 1970s, and the decline since then agrees with the observed decrease from 3 to 1.5 ng m^−3^ (EMEP 2016) in atmospheric Hg measurements (Fig. 2b). Modern atmospheric Hg concentrations of 1 to 1.5 ng m^−3^ remain elevated compared to estimates of Holocene, preindustrial Hg concentrations of 0.2 to 0.4 ng m^−3^ (Amos et al. 2013; Enrico et al. 2017; Li et al. 2020). The twofold decline in atmospheric Hg concentrations from the 1970s to 1990s is also reflected in declining atmospheric Hg deposition observations over that period (EMEP 2016), in natural peat archives of atmospheric Hg deposition (Fig. 2d, Li et al. 2020), and in the majority of biomonitoring studies that go back to the 1970s and 1980s (Fig. 2c, UNEP 2018). As can be seen in Fig. 2c and as recently discussed in the literature (Wang et al. 2019a, b; Grieb et al. 2020; Morris et al. 2022), not all biomonitoring studies show declines however, and the most recent global Hg assessment suggested that much of the variability in biomonitoring trends is related to regional Hg cycling complexity, and ongoing global change, including climate change factors (UNEP 2018). Over the period 1970 to 1990, bottom-up historical Hg emission estimates to air show either a similar decline from 3 to 2 Gg year^−1^ (Fig. 2a; Streets inventory) or an increase from 1 to 2 Gg year^−1^ (Muntean et al. 2018) due to varying underlying assumptions. The broad twofold decline observed in air, atmospheric deposition and biota has been attributed to environmental policy since the mid-1970s, aimed to curb industrial SO_2_ emissions that had measurable co-benefits on Hg emission and global Hg dispersal (Selin 2009).

Detailed, gridded primary anthropogenic Hg emission inventories have been developed for the 2000–2015 period (Muntean et al. 2018; UNEP 2018; Streets et al. 2019b; Fig. 2a). There is a good agreement among the three emission inventories suggesting an increase in annual Hg emissions mostly driven by the expansion of Asian economies. This recent increase in emissions is, however, in apparent contradiction with the observed decline of atmospheric Hg concentrations and Hg wet deposition in the Northern Hemisphere (Slemr et al. 2011; Weigelt et al. 2015; Marumoto et al. 2019; MacSween et al. 2022). This inconsistency might be due to the proximity of monitoring sites to regions where emissions have declined significantly in recent decades, e.g., continental Europe (Custódio et al. 2022), and North America (Zhou et al. 2017a, b). In that case, and as suggested by Lyman et al. (2020), comparison with regional or local emission inventories seems more appropriate to explain atmospheric trends. Other possible explanations for this inconsistency include changes in the speciation of emitted anthropogenic Hg (Zhang et al. 2016a, b) or global change effects such as enhanced vegetation Hg uptake due to a global increase in terrestrial primary productivity (Jiskra et al. 2018).

Figures 1, 2 and 3 illustrate that we have a reasonably complete understanding of the global Hg cycle and its main Hg emission and deposition fluxes today, and in the past. Industrial emission reductions in the 1970s and 1980s have resulted in measurable declines in Hg concentrations in Earth surface reservoirs and biota. Reductions in future Hg emissions from direct policy efforts under the Minamata Convention (Pacyna et al. 2016; Mulvaney et al. 2020), including indirect co-benefits from future shifts in energy consumption, i.e., less coal, will likely lead to further global reductions in atmospheric Hg concentrations and atmospheric Hg deposition to terrestrial and marine environments. Below, we discuss how global change may impact these Hg cycling and exposure trajectories.

## Global change and mercury cycling

**Fig. 4:**
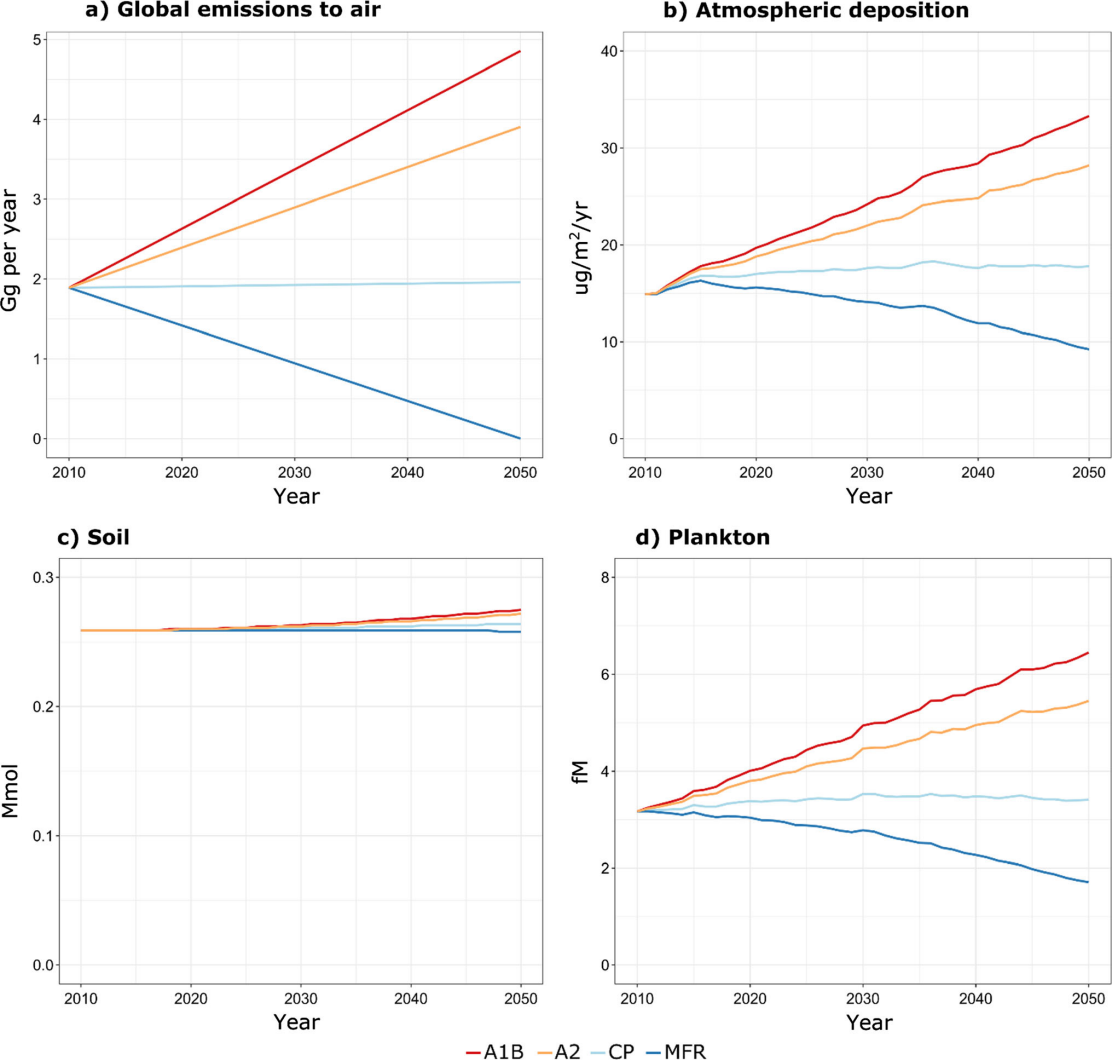
Projected global anthropogenic emissions and Hg levels in the environment in 2050 based on four scenarios. A1B and A2 refer to business-as-usual scenario or to a more fragmented economic growth and technological development, respectively. CP (current policy) and MFR (maximum feasible reduction) scenarios assume near-constant emissions or the application of the best available technologies, respectively. Adapted from Zhang et al. (2021a, b)

The Minamata Convention on Mercury entered into force in 2017, committing its current 140 parties to curb anthropogenic Hg emissions and release. Schartup et al. (2022) recently summarized projected global and regional anthropogenic Hg emissions into the future. Global anthropogenic emissions could decrease by up to 50% by 2035 if policy commitments and plans announced by countries worldwide are fully implemented [Fig. 4; maximum feasible reduction scenario (MFR)]. However, global emissions could double under a more fossil-fuel intensive A1B scenario. Future trends in ecosystem Hg thus strongly depend on the evolution of primary anthropogenic Hg emissions. Detecting a policy signal is, however, challenging because of the simultaneous impact of other global change factors on the global Hg cycle.

**Fig. 5:**
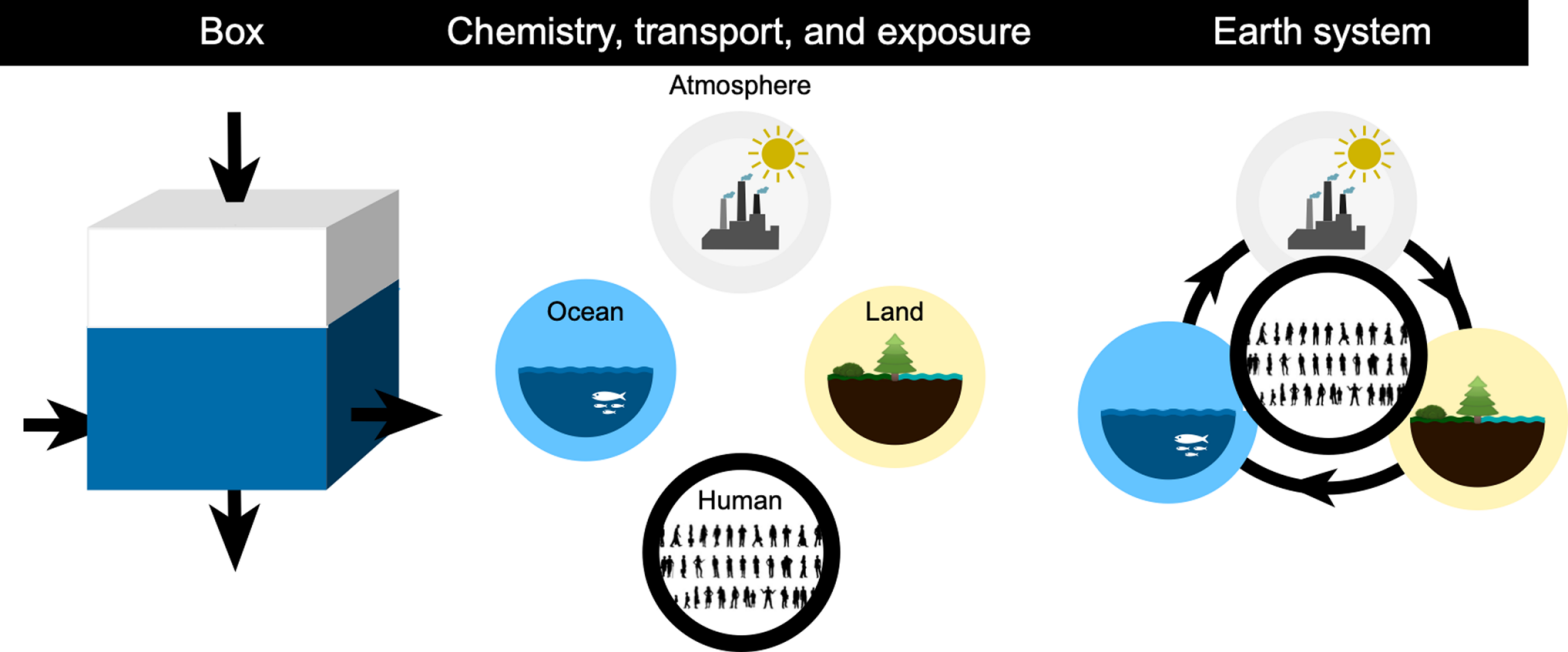
Schematic overview of different Hg modeling strategies (see also SI text). Box models (left) provide simple, flexible simulations of specific environments or the global Hg cycle, in particular over long, millennial time scales. Multi-dimensional chemistry, transport and exposure models (middle) provide more realistic simulations of regional or global Hg cycling that incorporate gridded emissions, 3D meteorology, biodiversity and ocean currents. Earth system models (ESMs) are comprehensive, coupled 3D models that allow simultaneous simulation of Hg emission, climate change and associated global change trajectories, including human and societal control factors

In its sixth assessment report (IPCC 2021), the Intergovernmental Panel on Climate Change (IPCC) states that human influence has warmed the atmosphere, ocean and land. This human-induced warming is already causing many weather and climate extremes in every region across the globe, including heatwaves, heavy precipitation, droughts, and tropical cyclones. Many changes due to past and future greenhouse gas emissions are irreversible for centuries to millennia, especially changes in the ocean, ice sheets and global sea level. Almost all aspects of global warming induced changes described in the IPCC (2021) report are affecting the Hg cycle. These changes are influencing Hg levels in aquatic ecosystems, biota, and ultimately human exposures, although the magnitude and direction of the effects vary. The combined effect of changes in emissions and climate is at the moment difficult to evaluate. Widely applied Hg emissions scenarios have, for example, not been built upon more recent global-scale climate change scenarios. As a consequence, future projections of anthropogenic Hg emissions are not directly comparable to projections of climate change (Schartup et al. 2022). A recent development in Hg modeling is to use comprehensive Earth system models (ESMs, Fig. 5) (Zhang and Zhang 2022). Unlike 3D transport models, ESMs can simulate the different spheres of the Earth surface system with unified suites of coupled modules. Another difference is the simultaneous simulation of the past and future climate, carbon, nitrogen, and phosphorus cycling, marine and terrestrial ecosystems, and even the human and society dimensions. In the following sections, simulation results for global change scenarios are therefore highlighted for a variety of model studies.

### Global change and atmospheric Hg transport and deposition

Atmospheric transport is one of the primary mechanisms by which Hg is distributed throughout the global environment. Climate change will, however, affect the transport of pollution from major emissions source regions. For example, by affecting mid-latitude circulation patterns, Arctic amplification (i.e., the disproportionate warming of the Arctic) impacts poleward pollution transport (Coumou et al. 2018; Chételat et al. 2022). In the absence of stricter mitigation policies, transport changes might also lead to increased surface pollution levels in the tropics due to reductions in vertical transport and dispersion by deep convection (Doherty et al. 2017). Hg deposition, the mechanism by which Hg enters ecosystems, depends on many different processes subject to change, including the magnitude of Hg emissions, circulation patterns, or land cover type.

Zhang et al. simulated changes in global Hg deposition from 2000 to 2050 under the IPCC A1B climate change scenario, which reflects integrated, rapid economic growth, and efficient use of technology and mixed energy sources (similar to RCP4.5, and recent SSP2 scenarios Zhang et al. 2016a, b). It was found that annual mean wet deposition flux increases over most continental regions, and decreases over most of the mid-latitude and tropical oceans. Land use and land cover changes lead to general increases in Hg^0^ dry deposition flux with large spatial variations due to the augmented Hg^0^ dry deposition velocity driven by changes in vegetation type and density. The combined effects of projected changes in climate, land use and land cover increased Hg deposition to the continental biosphere and decreased Hg deposition to the marine biosphere. At the global scale, the combined effects amounted to less than 10% change in deposition for the year 2050.

Vegetation uptake of atmospheric Hg^0^ is the most important Hg deposition pathway to the terrestrial environment (see budget section and Figs. 1, 3). Climate change and anthropogenic activities can influence the global Hg^0^ vegetation sink through multiple processes, e.g., deforestation, CO_2_ fertilization, vegetation species (biome) shifts, drought, ice storms, or fire frequency. Feinberg et al. (2022) recently optimized the Hg^0^ dry deposition parameterization in the GEOS-Chem model and simulated strong vegetation Hg^0^ uptake at the global scale (2300 Mg year^−1^), with the Amazon rainforest contributing 29% of the total Hg^0^ land sink. Continued deforestation and climate change (less precipitation over the Amazon) threatens the rainforest's stability and thus its role as an important Hg sink. In an illustrative worst-case scenario where the Amazon is completely converted to savannah, the GEOS-Chem model predicted that 400 Mg of Hg^0^ would not be sequestered in Amazonian soils annually, but mostly deposited to oceans. The physiological, climate and geographic factors controlling stomatal Hg^0^ uptake by foliage were recently investigated in a database of > 3000 European foliage samples (Wohlgemuth et al. 2022). Foliar stomatal Hg^0^ uptake varied with tree functional group, with deciduous leaves showing 3.2 times higher uptake than coniferous leaves, and foliar Hg^0^ uptake generally increased with nutrient (leaf nitrogen) and moisture availability. Global vegetation Hg^0^ uptake simulated with the GEM-MACH-Hg model (1700–2100 Mg year^−1^) gave estimates of annual Hg^0^ dry deposition fluxes to major global biomes that decrease from tropical broadleaf (26 μg m^−2^ year^−1^) to temperate broadleaf/mixed forests (18 μg m^−2^ year^−1^) to tropical grasslands (16 μg m^−2^ year^−1^) to temperate conifers (14 μg m^−2^ year^−1^) to temperate grasslands (9 μg m^−2^ year^−1^) and finally tundra (4 μg m^−2^ year^−1^) (Zhou et al. 2021). The ongoing northward shift in global biomes has been accompanied by an increase in net terrestrial primary production and potential net increase in vegetation Hg^0^ uptake of 140 Mg year^−1^ since 1990 (Jiskra et al. 2018). A new modeling study suggests that global reforestation policy could further stimulate Hg sequestration in soils and reduce Hg inputs to the ocean by 98 Mg year^−1^ (Feinberg et al. 2023).

### Global change and legacy Hg

Legacy Hg refers to Hg that was emitted in the past, deposited to land and oceans, and is still actively cycling and contributing to Hg re-emission, release to water, or assimilation and methylation by microbes. Important legacy Hg pools are present at contaminated sites (Rudd et al. 2021), in all global soils (Wang et al. 2019a, b), in coastal sediments (Reinfelder and Janssen 2019) and in the subsurface ocean (Amos et al. 2013). The burden of legacy Hg in the terrestrial environment (Figs. 1, 3), and in particular its slow movement through the soil–river–wetland continuum, suggests a potentially slower response of freshwater and coastal marine fish Hg levels to environmental Hg emission policy and a greater vulnerability to both subtle and extreme climate events. Remote lake sediment archives of, mostly, watershed Hg inputs have, for example, not shown significantly decreased Hg accumulation rates from the 1970s to 1990s in response to decreases in primary anthropogenic Hg release, emission and atmospheric deposition (Zhang et al. 2014; Amos et al. 2015; Li et al. 2020; Fig. 2d). The continued elevated Hg input from watersheds is likely the result of slow and continuous mobilization of legacy Hg from watershed soils, which may be subject to further increases or decreases in response to land-use change or wetter/drier climate. Land-use change and mismanagement such as deforestation or agriculture, can lead to enhanced soil erosion, mobilizing contaminated and soil Hg pools but also nutrients downstream to rivers, wetlands and coastal ocean. Below we illustrate these points by focusing on the Arctic region, where we see a confluence of both large legacy Hg pools and rapid climate change (AMAP 2021b).

Large pools of Hg have accumulated in Arctic soils (49 000 Mg for 0–30 cm depth; Olson et al. 2018; Schuster et al. 2018; Lim et al. 2020). While the Hg stock in the upper surface organic layers is mostly due to post-1960s atmospheric deposition, deeper mineral and permafrost soils store Hg that has accumulated over millennia (Olson et al. 2018). It is important to appreciate the size of the permafrost Hg reservoir, to predict its fate. The Arctic ocean contains approximately 1900 Mg of Hg (Petrova et al. 2020). The surface Arctic ocean mixed layer (0–20 m), where phytoplankton bioaccumulates MeHg, contains 44 ± 22 Mg of Hg (Soerensen et al. 2016; Petrova et al. 2020), which is a thousand times less than the 49 000 Mg in Arctic top soils. Warming induced changes in permafrost soil active layer depth, via enhanced soil erosion and organic matter leaching, may therefore mobilize sufficient Hg to dramatically impact Arctic Ocean Hg inputs and therefore marine biota MeHg levels.

Permafrost Hg mobilization may very well be of global importance. Arctic river Hg runoff is less dense than seawater, spreading out over the stratified surface Arctic Ocean. There, photoreduction of river Hg^II^ has been suggested to produce abundant gaseous dissolved Hg^0^ which is emitted to the atmosphere once sea-ice melts, potentially driving a 3-month long summertime peak in atmospheric Hg^0^ concentrations (Fisher et al. 2012; Zhang et al. 2015; Sonke et al. 2018). The global atmosphere is also a relatively small reservoir, containing 4000 Mg of Hg, compared to the 49 000 Mg in permafrost surface soils. Understanding what fraction of permafrost Hg will be mobilized to the atmosphere from soils, wetlands and the Arctic Ocean is therefore of global concern. A recent Hg isotope study suggests, however, that most river and coastal erosion Hg is buried in shelf sediments, without impacting the global atmosphere (Araujo et al. 2022).

Legacy Hg issues are not restricted to the Arctic. The sub-surface open ocean (100–1000 m) has accumulated large amounts of Hg over the past centuries by downward Hg transport during water mixing, deep water formation and via the biological particle pump, where surface water (0–100 m) primary productivity by phytoplankton generates biomass that binds or incorporates Hg and sinks to subsurface waters. After centuries of progressive Hg transfer from the atmosphere to the surface and to the subsurface open ocean, atmospheric Hg^0^ and Hg^II^ deposition has declined twofold since the 1980s (Fig. 2). Despite continued Hg transfer to the subsurface ocean by the particle pump, there is a non-negligible upward Hg flux sustained by turbulent mixing of subsurface waters into surface waters, in particular for MeHg (Zhang et al. 2020). This implies that the surface ocean Hg reservoir (2600 Mg) is buffered by the larger subsurface ocean reservoir (11 600 Mg) over decadal timescales. Consequently, global Hg models project a relatively slow recovery of surface ocean (Amos et al. 2015) and plankton Hg levels, e.g., a 25% decrease in plankton Hg for the year 2050, following a simulated 100% decrease in primary anthropogenic Hg emissions (Zhang et al. 2021a) (Fig. 4). Predicted ocean stratification under global warming may, however limit the upward supply of legacy Hg to the surface ocean and should be further investigated.

### Global change and Hg methylation

Organic Hg species are the link between the cycling of inorganic Hg described in detail above and the health concerns for biota and humans. Inorganic Hg conversion to methylated forms is primarily microbially mediated. Methylation occurs intracellularly through a corrinoid-binding protein (HgcA), a methyl carrier, and a ferredoxin (HgcB), acting as electron donor for reduction of the corrinoid cofactor (Parks et al. 2013; Cooper et al. 2020). Microbial Hg methylation is currently understood as an accidental process leading to the production and export of MeHg from the cell. Bioaccessibility and bioavailability of inorganic and organic Hg species to microbes depend on their partitioning onto solid phases and, in solution, depend on their association with chemical ligands. Together, these processes affect bioavailable Hg and MeHg species concentrations and how much MeHg enters the food web. Models that help predict the timing and magnitude of the pool of bioavailable Hg and MeHg, require variables that are sensitive to global change factors (e.g., variations in temperature, salinity, organic ligands, light penetration) that are reshaping elemental biogeochemical cycles. To identify drivers of Hg methylation, it is important not only to characterize Hg bioavailability as chemically defined species, but also to determine the functional dynamic of microbial players in the environment. An ecological framework describing the role of community interactions on the activity of microbes involved in organic Hg species transformation is currently lacking (Liu et al. 2019), nor do we yet have the data to build such a framework. What we can explore and mine for information, however, is the growing availability of microbial genomes which exhibit genetic determinants for Hg transformations. For this synthesis we explored such genomes with the goal to possibly link Hg transformations to the cycling of nutrients—which disruption by global change is well documented.

#### Functional insights into microbial Hg methylation and its relation to global change

**Fig. 6:**
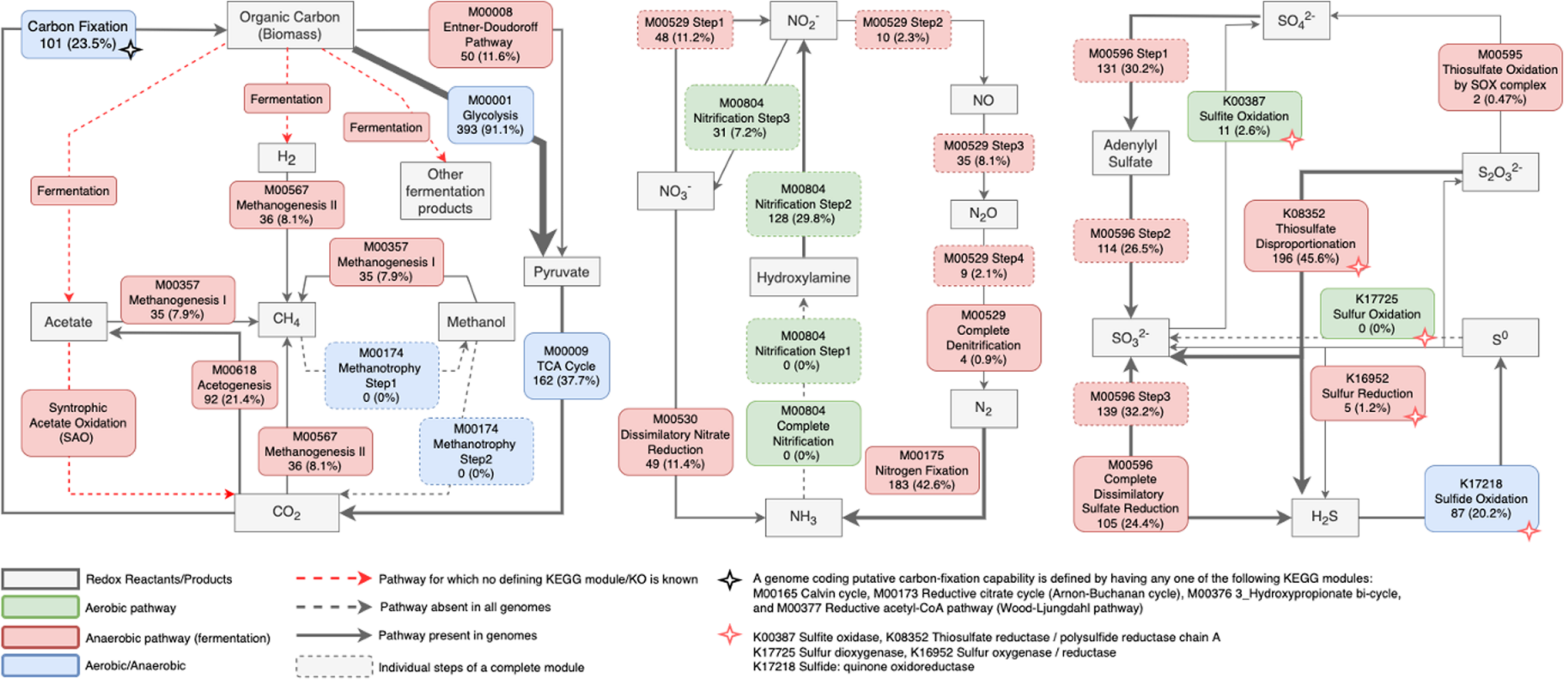
A diagram demonstrating the metabolic repertoire of Hg methylating microbes relating to the biogeochemical cycling of carbon, nitrogen, and sulfur compounds, with an emphasis on catabolic processes. This analysis revealed several potential coupling points between Hg methylation and microbial nutrient processing that are likely to be affected by global changes but with unclear direction and magnitude, and deserving further research. Notably, the net result of production vs. destruction of one-carbon compounds (e.g., CH_4_), or how alterations in nitrogen microbial metabolism (e.g., increased N_2_ fixation, or response to anoxia) will affect Hg methylation. For more information see the Supplementary Information

As our planet changes, microbial dynamics will be influenced by fundamental ecological interactions, such as resource accessibility, competition or predation; microbes will also become actors of these changes (Cavicchioli et al. 2019). For instance, provided with too little nutrients or affected by too much competition, a very potent mercury methylator identified in the lab, is unlikely to play a key role in the environment. Relying on genome annotations, we have summarized a current—yet incomplete—metabolic repertoire of Hg methylating microbes (i.e., those that carry *hgcA* and *hgcB* genes (Parks et al. 2013) relating to the biogeochemical cycling of carbon, nitrogen, and sulfur compounds, with an emphasis on catabolic processes (i.e., processes leading to energy conservation; here, we mostly omit anabolism or biosynthesis; Fig. 6). As a proxy for a potential Hg methylating cell, we refer to its genome reconstructed from sequencing environmental DNA, i.e., its Metagenome Assembled Genome (see SI for the full methodological approach, including QA/QC). A microbial cell whose genome contains the full set of genes required for Hg methylation is referred to as hgc+. Although incomplete, hgc+ repertoire exploration is useful to identify association between nutrients (C, N and S) and Hg species transformation pathways. The underlying hypothesis here is that disruption of nutrient cycling due to global changes (e.g., eutrophication) also affects the rate and magnitude of microbial Hg and MeHg transformation. This approach aims to identify potential coupling points between Hg and nutrient transformations that may be worth investigating. Only a few examples are offered here but we encourage the reader to perform the same analysis as genomic databases are populated.

Microbes affect major nutrient cycling. Increasing temperatures affect physical properties of water masses—fresh or marine—which in turn affect nutrient mobility and availability by increasing stratification. Land-use change affects nutrient mobility and availability via continental erosion and by the movement and physico-chemical composition of air masses, that control nutrient deposition to terrestrial and aquatic environments. With increasing water stratification, changes in overall primary productivity and altered atmospheric input, microbial nutrient acquisition and transformation will be affected. Oxygen minimum zones are expanding, favored by increased stratification, reducing oxygen availability and favoring the rise of anaerobic microbial metabolisms. Most Hg methylators are anaerobes that rely on the terminal oxidation of organic matter to produce energy, whether via fermentative or respiratory processes; these respiratory pathways include sulfate reduction and methanogenesis (Fig. 6). Any changes to the environment leading to greater anoxia and higher temperatures would be expected to stimulate organic matter oxidation—which final products are CO_2_ or CH_4_—and hence stimulate MeHg production. Methanotrophy (i.e., microbes consuming methane) is a major biological sink for atmospheric methane and its importance is expected to increase with an increase in the number of methane reservoirs in aquatic ecosystems (Mayr et al. 2020). No methylotrophs or methanotrophs were reliably identified as being mercury methylators. However, methanotrophs have been identified to conduct oxidative MeHg degradation (Lu et al. 2017), and a stimulation of methanotrophy by increasing temperatures and methane concentrations, may therefore not directly stimulate MeHg production, in fact it may enhance net MeHg degradation. The genomic data presented here support a need to predict future net MeHg production in the context of methane and other one-carbon compound cycling. Specifically, what would be the net result of MeHg production from methanogenesis once we account for MeHg degradation from increased methanotrophy? To answer such question, we first need to expand our knowledge of MeHg demethylation by methanotrophs and methylotrophs (Barkay and Gu 2022) and evaluate under which conditions methanotrophy can limit methane release from ecosystems (Mayr et al. 2020).

Pathways involved in nitrogen cycling have been found in *hgc*+ microbes (Fig. 6). The interplay between N and Hg cycling remains one of the least studied and possibly one that will require the most work in the coming years. The identification of *Nitrospina* as a widespread microorganism carrying and actively expressing *hgcAB* in mesopelagic oceanic layers (Tada et al. 2020; Villar et al. 2020; Lin et al. 2021) deserves further investigation in terms of its role in affecting net MeHg concentrations available to food webs. Members of Nitrospinae are aerobic chemolithoautotrophic microbes which derive their energy from the oxidation of nitrite into nitrate (Fig. 6). Nitrite oxidation is an important biological nitrate-forming reaction leading to bioavailable nitrogen species and this step has been found in 7.2% of the *hgc*+ microbes. Contrary to S cycling where intermediates (e.g., SO_4_) are directly involved in producing the energy required for Hg methylation, most intermediates in the N cycle are thought to be used in biosynthesis reactions. Our analysis also revealed that 10% of *hgc*+ microbes can denitrify NO_3_ to N_2_O (a potent greenhouse gas) with < 1% continuing the process to N_2_, and 11% reducing NO_3_ to ammonia (dissimilatory nitrate reduction to ammonia, DNRA). Denitrification, the process of using nitrate as terminal electron acceptor under limiting oxygen conditions, and typically leading to the production of gaseous N_2_O and N_2_, accounts for up to 50% of nitrogen loss from oceans to the atmosphere. We know very little of the direct role of nitrate reduction (whether it be denitrification or DNRA) on Hg methylation; in fact, nitrate amendments are often used to limit sulfate reduction, hence limiting MeHg production (Todorova et al. 2009). Furthermore, nitrate has a complex role in sulfate reducing bacterial activity (Green-Saxena et al. 2014), sometimes leading sulfate reducers to utilize nitrate when its presence in the community is not only a terminal electron acceptor competitor but also elicits a toxic response (Greene et al. 2003). Most surprisingly, almost half of the MAGs in the database show that *hgc*+ microbes have the genetic determinants for nitrogen fixation. The ability to fix nitrogen confers a competitive advantage, should nitrogen sources (e.g., NO_3_ or NH_4_) be or become limited, but could potentially lead to collateral increases in Hg methylation. In light of on-going global changes, the structure and function of microbial communities is affected in ways that net Hg methylation needs to be considered in the context of the presence of alternative electron acceptors, and of their role, beyond those used during sulfate-reduction, iron reduction or methanogenesis (Fig. 3).

Beyond the well-known sulfate reducers, the metagenomic analysis confirms that the interplay between S and Hg cycling is far more complex that currently described, and highlighted the role that multiple S intermediate transformations may have on Hg methylation. No supporting data currently exist to delve into the possible mechanisms associated with the genetic determinants revealed in Fig. 6, but it warrants additional work to clarify their potential to be environmentally relevant. Finally, most of the microbial pathways mentioned in this section rely on enzymes requiring metal cofactors as catalysts of their activity. Global change effects on MeHg cycling are likely subtler than merely stimulating a known pathway responsible for MeHg transformation. Indeed, such changes may also affect the availability of essential metals that are acting as enzymatic cofactors, hence indirectly affecting microbial metabolisms. In the presence of sulfide—a direct by-product of sulfate reducing bacteria activity—metal availability will change, and will likely decrease as most essential metals such as Zn or Cu, but also Co, Ni or Fe will form strong bonds with sulfides. Throughout its history, planet Earth has experienced great variations in the geochemistry of its environment (Anbar 2008) and recognizing that the rapid environmental changes that the planet is currently experiencing may affect the evolutionary path of microbial metabolisms must be kept in mind, when building predictive models.

### Hg^II^ availability for methylation

To predict the response of Hg^II^ methylation to global change, it is necessary to understand the biogeochemical controls on Hg^II^ bio-uptake and methylation. Formation of MeHg has been observed for contrasting dissolved, nanoparticulate, adsorbed and solid-phase Hg^II^ species added to natural soils, sediment, or water and to bacteria cultures (Schaefer et al. 2011; Jonsson et al. 2012; Zhang et al. 2012). The extent of methylation however differs substantially among Hg^II^ species, and the molecular mechanisms for Hg^II^ internalization and subsequent delivery to the active site of HgcA are not fully identified (Bravo and Cosio 2020). The current paradigm is based on experimental support for two types of uptake mechanisms: (i) diffusion, plausibly facilitated by porins, of sufficiently small and lipophilic complexes such as dissolved Hg^II^-sulfide species with neutral charge (i.e., Hg(SH)_2_^0^(aq)) (Benoit et al. 2001; Zhou et al. 2017a, b) and possibly nanoparticulate HgS species (HgS(np) (Graham et al. 2012; Zhang et al. 2012), and (ii) active uptake via metal transporters of several types of dissolved Hg^II^ species (Schaefer et al. 2011), including complexes with thiols and sulfide, and possibly HgS(np) species (Tian et al. 2021). It is likely that Hg^II^ uptake mechanisms partly differ among *hgc*+ microorganisms. Overall, experimental results support that the rate of uptake by diffusion increases with increased lipophilicity and smaller size of Hg^II^ species (Mason et al. 1996; Benoit et al. 2001; Zhou et al. 2017a, b), and the rate of active uptake increases with decreased thermodynamic stability and steric hindrance effects of Hg^II^ species (Schaefer et al. 2011; Adediran et al. 2019). These results are in accordance with generalized theoretical frameworks for bio-uptake of metals (Morel and Hering 1993; Hudson 1998; Zhao et al. 2016).

There has been a focus on dissolved Hg^II^-species with sulfide and with thiols in studies on MeHg formation. The concentration and exact composition of such Hg^II^ species are controlled by both thermodynamic and kinetic processes (Jonsson et al. 2012; Chiasson-Gould et al. 2014), the latter including redox cycling between Hg^II^ and Hg^0^ and time-dependent particle aggregation (Pham et al. 2014) and changes in crystal structure (Tian et al. 2021) of HgS(np) phases. Some types of Hg^II^-species span (or are expected to span) large ranges in availability for methylation depending on the exact chemical structure, in particular Hg(DOM-RS)_2_(aq) and HgS(np) species. Hg^II^ bioavailability can be operationally estimated for example by filtration, as the soluble Hg^II^ fraction, or as the stannous chloride reducible Hg^II^ fraction of a sample (Marvin-DiPasquale et al. 2014; Johnson et al. 2015). So far, diffusive gradients in thin films (DGT) measurements have been the most successful of these approaches to predict MeHg formation in environmental samples (Ndu et al. 2018; Neal-Walthall et al. 2022). Measuring the ligands that bind Hg^II^ also helps constrain Hg^II^ bioavailability estimates using thermodynamic and kinetic speciation models. A range of different thiol compounds, with a focus on cysteine, have been studied in Hg^II^ methylation experiments with bacteria cultures (e.g., Schaefer et al. 2011). A recent study, however, demonstrated that the thiols used in such experiments constituted only a minor fraction (~ 0.3%) of the total thiol concentration in boreal wetland porewater (Liem-Nguyen et al. 2021) meaning the chemical structure is unidentified for the vast majority of thiols and Hg^II^-thiol species. A better characterization of dominant thiols is thus needed to evaluate the role of Hg^II^-thiols for MeHg formation in such environments. An area that warrants further research is the potential role of Hg^0^ as substrate for MeHg formation in nature. Although the methylation rate was lower for Hg^0^ compared to Hg^II^-species in bacteria culture experiments (Colombo et al. 2013; Hu et al. 2013), it may very well be that Hg^0^ has a higher availability compared to the predominant Hg^II^ species in natural samples. Notably, Hg^0^ can be present in appreciable concentrations in soil and sediment pore waters following Hg^II^ reduction processes (Bouffard and Amyot 2009). A different, potentially highly important, aspect is microorganism-specific availability of Hg^II^ for uptake and methylation and how the composition of the entire *hgc*+ community controls Hg^II^ availability. It is clear that Hg^II^ uptake and methylation capacity can vary widely among microbes expressing *hgc* (Gilmour et al. 2011; Schaefer et al. 2011). Further, in nature the *hgc* genes are commonly found in high abundance in uncultivated organisms with unknown methylation capacity (Jones et al. 2019). The importance of the entire *hgc*+ microbial community composition for Hg^II^ methylation is thus unclear and it is possible that Hg^II^ availability in nature may change substantially due to hgc+ community shifts even without changes in Hg^II^ speciation or concentration.

Quantitative predictions of changes in MeHg formation and concentration due to global change are hampered by the fact that neither Hg^II^ speciation/availability nor *hgc*+ community composition are parameterized in current Hg cycling models. The reason for this lack is, as discussed above, the current insufficient fundamental understanding of how Hg^II^ availability and *hgc*+ community structure and activity quantitatively control Hg^II^ methylation. Here, we discuss in qualitative terms how different global change process can be expected to impact MeHg formation in the environment. Hg^II^ methylation rates determined from lab incubation experiments with pure microbial cultures or environmental samples span approximately two orders of magnitude among different dissolved Hg^II^ species, and another two orders of magnitude among different solid/adsorbed-phase species. There is thus potential for large changes in Hg^II^ availability in the environment if the biogeochemical conditions are altered, in particular regarding the cycling of S, Fe and C which controls the concentration of principal Hg^II^ ligands. Permafrost thawing has recently been highlighted as a process potentially leading to large regional increases in MeHg formation (Schaefer et al. 2020; Tarbier et al. 2021). The increase is driven by increased microbial activity and solubility of Hg^II^ following soil thawing. In the ocean, formation or concentration of MeHg have been linked to the rate of carbon mineralization and/or dissolved oxygen concentration (Cossa et al. 2009; Sunderland et al. 2009). Expansion of oxygen-deficient and anoxic zones, and increased rates of carbon mineralization are expected in the oceans and coastal seas, driven by increased temperature and nutrient inputs (Breitburg et al. 2018). These processes are expected to increase MeHg in parts of the global ocean (Booth and Zeller 2005; Zhang et al. 2021b). Development of oxygen-deficiency/anoxia will lead to increased activity of *hgc*+ microbial communities and increased solubility and availability of Hg^II^, in particular under sulfidic conditions with a predominance of dissolved Hg^II^-sulfide species over HgS(s) (Capo et al. 2022). Development of euxinic conditions in coastal zones due to high primary production is thus expected to have potentially large consequences on MeHg formation in the water column. To what extent MeHg formed in euxinic waters is incorporated in the pelagic food web is however less clear (Soerensen et al. 2018). A recent study also examined the impact of changing meteorology and ocean physics, including wind speed and seawater temperature on the size of Hg^II^ pool in the surface ocean, and found an overall offset effect in a hypothetical twenty first century ocean (Wang et al. 2023). They also found that the changing light environment plays a more important role in determining MeHg concentrations due to their sensitivity to photodemethylation processes. In terrestrial systems that will be subjected to increased rainfall, stronger soil redox fluctuations are expected to occur, which commonly lead to increased MeHg formation (Creswell et al. 2008; Eckley et al. 2015; Zhu et al. 2018). At the initial flooding *hgc*+ microorganisms can access and utilize increased concentrations of electron donors (organic matter) and acceptors (e.g., sulfate and ferric iron) which may be close to depleted under strict anoxic conditions. Subsequent decrease in the water table and re-oxidation events can restore the concentration of electron acceptors and also alter the relative concentrations of strong ligands (i.e., reduced sulfur compounds) in the aqueous and solid/adsorbed phases (Zhu et al. 2018). These examples illustrate the need for improved parameterization of both Hg^II^ availability and composition and metabolic activity of the hgc+ microbial community to quantitatively predict changes in MeHg formation following critical global change processes.

### Global change and MeHg in food webs

As the planet changes, animals are forced to respond to new environmental stressors that may impact their survival. We are witnessing these adaptations and behavior changes globally, such as reduced growth, changes in preferred prey, selection of new habitats, or hunting strategies (Rijnsdorp et al. 2009). Since MeHg accumulation in food webs occurs primarily through diet, adaptations and behavioral changes that influence the flow of energy also impact MeHg movement through food webs. While biomagnification of Hg in aquatic food webs is well characterized, descriptions of how recent animal adaptations and changing behavior modulate MeHg’s transfer is less well understood. Yet this understanding is important for predicting exposures for biota and humans, particularly as anthropogenic Hg inputs decline. Here we provide a few examples from the literature of how shifts in hydrology or land use, species introduction (invasion), or removal (fishing) can impact Hg in food webs. We also explore how climate change—through changes in temperature, sea ice, primary production, bioenergetics, and food web structures—impacts MeHg biomagnification.

A good predictor of Hg levels in the food webs is the concentration of aqueous MeHg in the ecosystem (Wu et al. 2021; Zhang et al. 2021b; Blanchfield et al. 2022). It means that any change to Hg loadings or transformations in an ecosystem will impact MeHg accumulation in biota. Blanchfield et al. (2022) and Wu et al. (2021) directly link water MeHg concentrations to MeHg in fish. This finding is consistent with surveys and statistical approaches in the marine environment that show that tuna Hg (i.e., MeHg) (Tseng et al. 2021; Barbosa et al. 2022; Medieu et al. 2022) is related to seawater MeHg. Theoretical and modeling approaches also estimate that seawater MeHg levels account for much of the MeHg variability observed in fish (Alava et al. 2018; Schartup et al. 2019). The implication of these findings is that if MeHg declines in a system then concentrations in biota will follow. Blanchfield et al. also report that lake food webs can partly recover if direct atmospheric Hg inputs decline precipitously. They show that MeHg quickly decreases by up to 91% in lower trophic level organisms. However, the decline in the large-bodied fish population was expectedly lagging with a decrease of 38–76% of MeHg 8 years after Hg loading ceased. These findings confirm that changes that impact the environmental levels of MeHg will have a cascading influence on food web MeHg levels and that efforts to reduce MeHg in the environment should remain a priority for policymakers.

Anthropogenic activities primarily release inorganic Hg, a fraction of which is converted to MeHg through natural microbially mediated processes. Conditions at the site (e.g., presence of organic matter, oxygen levels, microbial communities) control the amount of MeHg produced and entering the food webs. Hydrology and land use have a direct impact on these site conditions (Buckman et al. 2021). A land use change that is of great importance for Hg is river impoundment and reservoir creation which often result in an increase in MeHg production and accumulation in biota following the decomposition of soil organic material. Some studies report a three- to sixfold increase in predatory fish Hg levels in new reservoirs (St. Louis et al. 2004; Calder et al. 2016). The impact on biota MeHg concentration can be substantial, even when MeHg levels in the ecosystem do not change (Hsu-Kim et al. 2018). Walters et al. studied the food web assemblages and Hg distribution before and after an experimental flooding event in the Colorado River (Walters et al. 2020). They compare a simple inefficient food web immediately below a dam, easily perturbed by flooding events, with a downstream food web that is more complex, efficient, and resistant to flood perturbation. They find large differences in Hg transfer between the two food webs and concluded that land use changes can influence Hg levels in food webs by modifying the species present and their interactions.

The increasingly recognized role of vegetation in the uptake and sequestration of Hg^0^ (Figs. 1, 3) means that we need to pay attention to its role in transferring Hg to terrestrial and aquatic food webs. In terrestrial food webs, plants and associated insects from the bottom of food webs are an important conduit of Hg. Hg accumulates in terrestrial food webs similarly to aquatic ecosystems, with the lower trophic positions dominated by inorganic Hg, and the fraction of MeHg increasing with the trophic position. Li et al. (2021) discuss biomagnification in a plant–insect–spider–songbird food chain of a remote subtropical forest ecosystem (Li et al. 2021). And like in aquatic systems, they find that life traits such as habitat, trophic guild, feeding guild, and diet at both the larval and adult stages significantly influence the Hg accumulation in tissue. Yung et al. (2019) studied total Hg and MeHg accumulation at a contaminated phytomanaged site and an adjacent control forest site (Yung et al. 2019). Insect Hg levels were ten times higher in the contaminated than control site. But when they compared insects living on plants (nettle) to insects living elsewhere, the nettle food web had 6 times lower Hg levels than insects not living on nettle. They also found that the nettle-unrelated insect habitat was more important than diet, i.e., contact with contaminated soil and water. This study suggests that the presence of vegetation can insulate food webs from contamination. But the role of vegetation varies; in their study Chiapella et al. (2021) report that near-lake tree cover accounted for a large portion of lake fish Hg. They show a positive association between the presence of conifer and fish Hg and posit that this is due to tree accumulation of Hg and deposition of needle Hg near lakes (Chiapella et al. 2021). The study concludes that shifting tree lines—from deforestation, reforestation or climate change—could impact Hg bioaccumulation.

As shown in the examples above, changes in biological species interactions can influence Hg transfer in food webs and either increase or decrease the Hg burden for top predators. We can infer that introducing or removing species through invasion, overfishing or management practices can impact MeHg levels in food webs. Several examples in the literature confirm this inference. Lepak et al. investigated MeHg accumulation in Lake Michigan predatory fish (Lepak et al. 2019). They found that despite a substantial reduction in Hg inputs since the 1970s, MeHg level in lake trout did not decline during their study period (1978–2012). They attribute this result to the arrival of invasive mussels and their predators that forced the food web to rely more heavily on benthic energy sources and legacy Hg. Seco et al. (2021) made similar observations in the Southern Ocean, where they observed rapid (interannual) shifts in preparatory birds' Hg levels in response to diet shifts (Seco et al. 2021). They found that the reduced availability of a low Hg key prey, the Antarctic krill *Euphausia superba*, during the 2016/2017 sampling period forced seabirds to rely on higher trophic level (and thus higher Hg) prey compared to 2007/2008. The studies illustrated highlight how natural and global change factors affect food web structure, which in turn influences top predator MeHg exposure. In the next section we look at recent modeling and field studies that predict changes in Hg cycling and biota exposure.

### Projected changes in global Hg cycling and implications for biota exposure

The impact of global change on MeHg accumulation in food webs involves multiple simultaneous forcings with mixed effects on the overall MeHg levels in seawater and biota. For instance, in the Arctic, increasing temperatures melt sea ice—an important ecological habitat—thus restructuring the food web (AMAP 2021a). But the decline in sea ice also impacts Hg air–sea exchange and exposes marine MeHg species to more sunlight, potentially removing MeHg from surface waters. Warmer temperatures accelerate permafrost thaw, increase freshwater discharge and the associated nutrient inputs, resulting in higher terrestrial Hg export to coastal areas and potentially enhanced primary production (Vancoppenolle et al. 2013). Quantifying the overall result of these changes on Hg methylation and biological uptake is challenging. Modeling and experimental approaches can help disentangle these forcings and project trends. Schartup et al. (2022) modeled the relative response of Hg in the Arctic Ocean to policy scenarios, changing sea-ice cover, river inputs, and net primary production. The authors found that in the medium-term (2050) anthropogenic Hg emissions exert a stronger influence on the Arctic Ocean Hg levels than climate-induced effects. However, the study did not consider permafrost thaw, Hg methylation, MeHg uptake, or food web structure.

A study by Zhang et al. (2021a) estimated the global health effects of future atmospheric mercury emissions using a comprehensive climate–atmosphere–land–ocean–ecosystem and exposure-risk model framework for Hg. They found that future global atmospheric Hg deposition and marine planktonic MeHg are sensitive to global Hg emission reductions (Fig. 4), consistent with the conclusions of Schartup et al. (2022) for the Arctic. Zhang et al. compared predicted global Hg deposition for the CP scenario to the MFR scenarios. MFR scenario Hg deposition was 48% lower, even though the corresponding global total anthropogenic emissions are 85% lower. This is due to the effect of legacy emissions that contribute ~ 60% of total annual global Hg emissions (Amos et al. 2013). The percentage change projected for the marine planktonic MeHg concentrations is similar to atmospheric deposition, while the changes in soil Hg concentrations are much smaller due to the large mass and long lifetime of soil Hg. Similarly, the A1B and A2 emission scenarios also drive an increase in atmospheric deposition and ocean Hg levels, even though the magnitudes of changes are smaller than that of anthropogenic emissions. This study calculates a cumulative economic loss of $19 trillion USD for the period 2010–2050 (discounted to 2050) for the CP scenario, while the MFR scenario can reduce the loss (i.e., a benefit compared to the CP scenario) by $2.4 trillion USD and the A1B and A2 increase the loss by $4.9 and $3.3 trillion USD, respectively. Zhang et al. (2021a) reported global mean changes and did not discuss regional changes in detail. It is known however that unique regional responses are happening due to the rapidly changing conditions in coastal environments with rapid eutrophication, and in polar regions with thawing permafrost and melting sea ice. Future improvements to the modeling framework could also include a better interaction between climate-driven impacts, food web structures and Hg cycling. The study is thus a good starting point for further investigations that involve more realistic changes in the global and regional environments that are important for Hg cycling.

Schaefer et al. (2020) estimated the impacts of permafrost Hg release on fish MeHg levels for the Yukon River basin using a mechanistic land surface model that incorporates coupled carbon, water, energy, and Hg cycling. They showed that under the high greenhouse gas emissions scenario Representative Concentration Pathway (RCP) 8.5, Yukon River fish Hg levels may exceed US EPA fish guidelines of 0.3 µg g^−1^ by 2050 (Schaefer et al. 2020). But this study did not consider changes in food web structure or bioenergetics. Others developed and applied a trophodynamic ecosystem model to project Hg accumulation under two climate scenarios, RCP 2.6 and 8.5 (Alava et al. 2018). They predicted higher MeHg levels in salmon and killer whales under a high carbon emission scenario, with a 10% increase MeHg in salmon and an 8% increase in killer whales. Schartup et al. (2019) tested the relative influence of temperature, seawater MeHg concentrations, and diet shifts using a bioenergetic model applied to the Gulf of Maine. They found that while seawater temperature increase can lead to higher fish MeHg levels, the overall response to changing ecosystem conditions and prey availability results in contrasting effects on MeHg concentrations across species. A simulation of the combined impact of a decline in herring fish, an increase in seawater temperature, and a decrease in seawater MeHg on cod and dogfish showed an opposing response in MeHg levels. Simulated tissue MeHg concentrations in cod in the 1970s were 10% lower than for cod consuming a diet typical of the 2000s that relied more heavily on large herring, lobster, and other macroinvertebrates. While the 1970s diet for spiny dogfish, when herring was unavailable, included a higher proportion of squid and other cephalopods, resulting in 60% higher MeHg in the 1970s than in the 2000s in spiny dogfish. In a global simulation of MeHg in marine plankton, Zhang et al. (2021b) shows that phytoplankton MeHg may increase at high latitudes and decrease in the mid- and low-latitude oceans due to the shifts in phytoplankton communities driven by climate change. Ocean acidification could enhance MeHg bioaccumulation by promoting the growth of small phytoplankton species that efficiently accumulate MeHg. But how phytoplankton distribution and uptake will impact higher trophic levels remains uncertain since the study also revealed that patterns of zooplankton MeHg accumulation differ from phytoplankton due to complex grazing relationships.

Field based studies sometimes, but not always, align with the modeling and experimental results. Dietz et al. (2021), used 10 narwhal tusks in Northwest Greenland and measured their isotopic (N and C) and Hg content to derive climate-induced dietary shifts and the influence of sea ice loss on Hg (Dietz et al. 2021). The study showed a log-linear increase in Hg between 1962 and 2010, with a rapid rise in recent years and proposed that this recent rise is due to the accelerating decline in sea ice and/or greater bioavailability of MeHg. In a 2018 “space for climate substitution study”, Ahonen et al. collected six fish species from 18 European subarctic lakes across a climate gradient (Ahonen et al. 2018) and found that Hg tissue concentrations are higher in warmer and more productive lakes. They also found that nutrient inputs (N and P) affect Hg levels although the source of nutrient inputs from sewage and or agriculture or forestry had an opposing impact on fish Hg levels, with sewage/agriculture decreasing and forestry activities increasing Hg in fish. A similar sampling concept was applied to Canadian lakes (Sumner et al. 2020), where no long-term impact of temperature or precipitation on current muscle Hg was found in the fish species studied. A marine study in the Peruvian upwelling zone examined if ENSO-induced variability in marine biogeochemistry affected blood Hg concentrations in marine seabirds. Despite sea surface temperature anomalies up to 3 °C, oxycline depth change from 20 to 100 m, and strong primary production gradients, no measurable trend in seabird Hg concentrations was observed between el Nino, la Nina, and normal years. The profound impact of ENSO on lower trophic food web ecology, e.g., 10 × variability in anchovy biomass, visibly does not lead to major changes in MeHg exposure to marine top predators. Jonsson et al. used a series of mesocosm model experiments as an intermediate approach between field and modeling approaches where sediment, water and biota conditions, and MeHg levels and sources could be controlled. They demonstrated that in coastal/estuarine ecosystems, climate change-driven increases in terrestrial organic matter and nutrient loads resulted in a substantial increase (two- to sevenfold) in biota MeHg levels due to a combination of enhanced MeHg availability and lengthening of the food chain (Jonsson et al. 2014, 2017).

In summary, numerical models that capture the complexity of real-world Hg and MeHg cycling, ecodynamics and human exposure have made considerable progress over the past decade. It is imperative that these models are calibrated against a variety of long-term monitoring trends of environmental and biota Hg and MeHg concentrations, before their predictions can truly inform environmental policy making.

## Conclusions and perspectives

In this synthesis paper we summarized the state of the science regarding global change impacts on Hg cycling. Changes in Hg release and emission trajectories are part of global change and are closely linked to the energy sector and to mining. Past environmental policy since the 1970s has had a measurable, positive impact on environmental Hg levels that have decreased broadly by a factor of two. Successful curbing of new Hg emission and release under Minamata Convention policy will therefore likely lead to further declines in environmental Hg levels and human exposure. Global change impacts may reinforce or offset policy efforts in ways that are difficult to predict today. The studies we highlighted suggest the following: atmospheric Hg transport and deposition, including dominant vegetation uptake, are sensitive to meteorology and biome shifts. Modeling studies suggest that future Hg deposition and re-emission patterns will change substantially at the regional level, but not globally. Biome shifts, deforestation, soil erosion, reservoir creation, permafrost thaw will impact Hg cycling and exposure in downstream ecosystems, but again, the net effect is presently hard to predict. Legacy Hg pools at contaminated sites and in global soils can be rapidly mobilized during extreme weather (floods, fires) and climate events (permafrost thaw, glacier melt). We expect that an additional twofold, or larger, decrease in Hg emissions and release under the Minamata Convention should be distinguishable from climate change effects at the regional and global scales, despite their inherent uncertainties. Global change effects on Hg cycling and exposure at the local scale may however be larger than impacts from Hg release policy. This has implications for Hg monitoring programs that assist in the effectiveness evaluation of the Minamata Convention. Well mixed reservoirs, such as the atmosphere and subsurface ocean below the photic zone, but also major rivers that integrate continent-wide Hg dynamics, are more likely to detect robust trends that inform on policy vs. global change effects. Similarly, top predators in aquatic food webs integrate a cascade of complex MeHg production, breakdown, and trophic transfer factors and are therefore more likely to detect trends that are difficult to observe in lower trophic species. Alternatively, detecting long-term trends under highly dynamic conditions and strong Hg variability associated with redox chemistry in water, air, vegetation or with plankton dynamics and grazing is possible but generally requires more observations both in time and in space. In addition to monitoring programs, next-generation ESMs can also assist in the effectiveness evaluation. By simulating past and future global change, they can help decouple anthropogenic and climate-driven signals on multiple spatial and temporal scales.

Microbial genomes and environmental metagenomes analyses have revealed that the genetic determinants necessary for mercury methylation can be found in diverse environments spanning a large range of redox conditions, from oxic to anoxic systems. These genes appear to remain mostly associated with anaerobic microbes, which can thrive in anoxic microniches present in heterogeneous oxic aqueous systems. The presence of Hg methylation genes in aerobes is intriguing. The environmental relevance of aerobic Hg methylation remains to be tested experimentally. As we gain functional insights into Hg methylation at the microbial cell, population, and community levels, it becomes essential to evaluate how disruption of nutrient cycling due to global change, will also affect the rate and magnitude of microbial Hg and methylated Hg species transformation and bio-uptake of Hg species.

### Monitoring and research needs

Historically, Hg monitoring programs have focused on key parameters that were technically straightforward to measure: atmospheric Hg concentrations, Hg^II^ wet deposition and biota total Hg concentrations (including human biomonitoring). In the introduction we have highlighted recent research that shows (1) gaseous Hg^0^ deposition, by vegetation and ocean uptake, to be more important than Hg^II^ deposition, and (2) Hg release to water and land to surpass Hg emissions to air. Major Hg release and ecosystem loading pathways are therefore unmonitored, or even unmeasured due to technical challenges. Monitoring guidance for the effectiveness evaluation of the Minamata Convention therefore still focuses on atmospheric, biota and human tissue monitoring. Because global change is likely going to modify key Hg release and transformation processes at the local and regional scales we recommend additional monitoring efforts across all climate zones, keeping in mind feasibility issues:

- Total Hg, and where possible MeHg, concentrations in major regional rivers and their tributaries. River runoff integrates complex global change processes at the terrestrial watershed scale such as land-use and flooding and is the dominant Hg source to coastal ecosystems. Monitoring of river biogeochemistry and Hg, such as done by ArcticGRO (great rivers observatory; https://arcticgreatrivers.org/), also provides critical information on nutrient transport, erosion, and hydrology.
- Coastal sediment and water total Hg and MeHg concentrations. Coastal fisheries represent the largest MeHg exposure source to humans globally (Lavoie et al. 2018), surpassing open ocean catch. Coastal seafood biomonitoring is sufficiently covered by monitoring programs, yet to understand biomonitor trends and deconvolute biogeochemical and ecological global change factors the Hg and MeHg concentrations in coastal water and sediment need to be monitored.
- Hg flux measurements at the land–air and water–air interface. Novel, near-surface, snow and soil air atmospheric Hg^0^ gradient methods, and eddy covariance flux methods have shown promise in determining whole ecosystem net Hg^0^ exchange data, ranging from net deposition to net emission at diurnal and seasonal time scales. On land, Hg flux monitoring should be collocated with existing climate monitoring sites (e.g., US NSF LTER program, eLTER, ICOS, etc.), where decades of climate and biota data such as temperature, CO_2_, O_2_, nutrients, or microbial community structure are already available.

At a higher technical level, we recommend monitoring efforts to explore Hg isotope signature and omics analyses. Hg isotopes have been instrumental in identifying and quantifying the importance of vegetation Hg^0^ uptake (Enrico et al. 2016; Zhou et al. 2021), and provide at all times additional information on changing Hg sources or transformation pathways (Kwon et al. 2020). We expect genomics and metabolomics analyses to be particularly relevant for global change research and monitoring as it provides insight on the single most important reaction in the Hg cycle, methylation.

ESMs have become a key tool to investigate and understand global change and climate change effects on Hg cycling, both at the global but also at the regional level. Earth system modeling efforts, using shared community software, should be expanded and continued and intensified interaction between naturalists, experimentalist and modelers is needed to efficiently address knowledge gaps. ESM diversity, exploring different parameterizations for the same processes are essential to evaluate modeling uncertainty, via intercomparison exercises and modeling ensemble approaches.

The Arctic is currently warming three times faster than other global regions, leading to dramatic ecosystem changes. Arctic permafrost top soil (0–30 cm) stores ~ 10 times the amount of all anthropogenic Hg emissions over the last 30 years, a stock that will be partly remobilized to air and aquatic ecosystems due to climate change-induced permafrost thaw. While recent work suggests that most Arctic river Hg is currently buried in shelf sediments (Araujo et al. 2022), i.e., does not enter the global atmospheric Hg pool, significant regional impacts can still be expected with consequences for local communities that rely on fresh water and marine fish. Other climate-related Hg remobilization pathways include increased wildfire frequency or melting of the Greenland ice cap (Dastoor et al. 2022). As there is a confluence of both large legacy pools and rapid climate change, the Arctic is an ecosystem sentinel for regional and global change. As such, continued monitoring and modeling efforts in this region are warranted.

A last major area that needs further research is MeHg formation in oxic waters, in particular in the ocean, including a focus on Hg^II^ speciation and bioavailability under oxic conditions. The processes for MeHg formation under such conditions are not yet clear and several have been suggested (Gascón Díez et al. 2016; Gionfriddo et al. 2016; Bowman et al. 2020; Wang et al. 2022): (i) *hgc*-driven Hg^II^ methylation by obligate anaerobes in anoxic microenvironments; (ii) *hgc*-driven Hg^II^ methylation by microorganisms that are not obligate anaerobes; (iii) Hg^II^ methylation driven by other microbial pathways in aerobic microorganisms; and (iv) Hg methylation by abiotic processes. If we want to fully understand and anticipate global change effects on Hg methylation and bio-uptake in marine ecosystems we must first identify the dominant methylation mechanisms and control factors on Hg^II^ and MeHg speciation. In order to parameterize Hg^II^ and MeHg speciation in ESMs continued research efforts are key.

## Supplementary Information

Below is the link to the electronic supplementary material.

Supplementary file1 (PDF 636 kb)
