# Supplementary material for: Global change effects on biogeochemical mercury cycling
Source: Ambio. 2023 Mar 29;52(5):853–76. doi: 10.1007/s13280-023-01855-y (PMC10073400; doi:10.1007/s13280-023-01855-y)
Supplement: Supplementary file 1 — Supplementary file1 (PDF 636 kb) [file 13280_2023_1855_MOESM1_ESM.pdf]

Ambio
Electronic Supplementary Material

Title: Global change effects on biogeochemical mercury cycling

Authors: Jeroen E Sonke, Hélène Angot, Yanxu Zhang, Alexandre Poulain, Erik Björn, Amina T Schartup

Earth System models

Earth system models combine physical transport and biogeochemical Hg transformations in different spheres (e.g., atmosphere, hydrosphere, lithosphere, and biosphere) of the Earth and are important tools for predicting future changes in global and regional Hg cycling, and human exposure. There have been three major stages in Hg model development for Hg (Fig. 5): 1) 0D and 1D box models, where complex systems are simplified to boxes/reservoirs linked by fluxes; 2) 1D/2D/3D chemistry transport models with Hg biogeochemistry that typically focus on one or two spheres; and 3) Earth system models that couple all the major spheres of the Earth. Early efforts to model the global Hg cycles include those by (Mason et al. 1994) and (Lamborg et al. 2002), in which the ocean is divided into 2-3 boxes based on depth while the atmosphere is divided into two hemispheres. Later improvement includes the work by Sunderland and Mason (2007) that considered the transport of Hg across different ocean basins and those by Amos et al. (2013) and Amos et al. (2014) addressing historical Hg release. Strode et al. (2007) developed the first 1D and 2D models for ocean Hg, coupled to a 3D atmospheric model. Three-dimensional oceanic models were first developed by Zhang et al. (2014a) in the HIM-GOLD model and later other models such as MITgcm (Zhang et al. 2015), NEMO (Semeniuk and Dastoor 2017), and FATE (Kawai et al. 2020). Three-dimensional models for the atmosphere were developed in the late 1990s, including the GISS-CTM (Shia et al. 1999), GEOS-Chem (Selin et al. 2007), GRAHM (Dastoor and Larocque 2004), CMAQ (Bash 2010), and CAM-Chem (Lei et al. 2013). The model development for Hg in the soils and vegetation has been slower compared with atmosphere and ocean with the first 2D mechanistic terrestrial Hg model (GTMM) developed by (Smith-Downey et al. 2010) based on the CASA soil carbon dynamic model. A recent extension that includes the vertical structure of permafrost (SiBCASA) was achieved by Schaefer et al. (2020).

Dynamic/online coupling of these 3D models for different spheres (mainly atmosphere, ocean, and land) has also been achieved, including the atmosphere and ocean (Zhang et al. 2019) and atmosphere-land-ocean (Zhang et al. 2021b). These models have been successful in interpolating observed spatial patterns of observations, such as the inter-hemispheric atmospheric Hg gradient and ocean Hg vertical profiles, testing mechanistic hypotheses, such as atmospheric redox chemistry and ocean Hg methylation, up-scaling local observations to global scales, such as the air-sea exchange flux and vegetation uptake, and making predictions, such as the impact of climate and land use/cover change on atmospheric Hg (Zhang et al. 2016; Zhou et al. 2021; Feinberg et al. 2022), and ocean warming and acidification on ocean Hg (Zhang et al. 2021b).

A recent development in Hg modeling is to use comprehensive Earth system models (ESM) (Zhang and Zhang 2022). Unlike 3D transport models, ESMs can simulate the different spheres of the Earth surface system with unified suites of coupled modules. Another difference is the simultaneous simulation of the past and future climate, C/N/P cycling, marine and terrestrial ecosystems, and even the human and society dimensions. There are thus several major advantages of ESMs: 1) all the environmental media are coupled in a consistent and mass-balanced manner, which can be difficult to achieve by coupling models with different resolution and physical assumptions; 2) it is easier to investigate the impact of future climate and ecosystem changes, which are typically the major aims of ESMs; 3) it is an ideal tool to test the synergy of multiple policies involving carbon neutralization, eutrophication, and the mitigation of other pollutants (such as riverine nutrient discharge, atmospheric emissions of particulate matter, ozone, and their precursors); 4) the incorporation of human and society components in ESMs also facilitates the evaluation of human MeHg exposure via diet and other pathways (e.g., (Zhang et al. 2021a)) In the following sections, simulation results for global change and climate change scenarios are highlighted for a variety of model studies.

Methodology used to develop the *hgc*+ Figure 4:

We leveraged the recently compiled Hg-MATE database (Gionfriddo et al. 2021; Capo et al. 2022) and conducted a metabolic analysis to investigate the potential role that mercury methylating microbes play in global nutrient cycling. First, we selected the methylators in the database with an NCBI genome ID and removed the genomes containing the fused hgcAB or lack either an HgcA or HgcB sequence, then

downloaded the DNA sequences of the genomes that met the selection criteria from the NCBI assembly database. The quality of the resulting genomes were examined using CheckM (v1.1.3) (Parks et al. 2015), and we retained genomes with over 80% completeness and less than 5% contamination for downstream analyses. We determined the taxonomy of the genomes using GTDB-tk (v1.4.1, GTDB rs202) (Chaumeil et al. 2020; Parks et al. 2020), then removed duplicated species and retained only one representative species with the highest completeness value. To analyze the metabolic potential of the selected methylator genomes, we used Anvi'o (v7.1). Briefly, we 1) used `anvi-gen-contigs-database` to calculate k-mer (i.e., 4) frequencies in each genome and identify open reading frames (ORFs) using Prodigal (v2.6.3) (Hyatt et al. 2010), 2) annotated the ORFs and assigned them KEGG orthologs(KO) numbers with `anvi-run-kegg-kofams` against the Kofam HMM database (Aramaki et al. 2020), 3) estimated the completeness of various KEGG modules (Kanehisa et al. 2016) using `anvi-estimate-metaoblism` across all genomes.

We classified the methylator genomes into various metabolic categories based on their source of carbon (autotroph/heterotroph), energy(chemotroph/phototroph) electron donor (organotroph/lithotroph) and terminal electron acceptor (aerobe/anaerobe (including fermenters)), inferred based on the presence or absence of corresponding KEGG modules coding the corresponding traits. A module is considered present in a genome when the completeness of the module is greater than 75%, and absent when below this threshold. Specifically, an autotroph is defined by having one of the following modules, including the Calvin cycle (M00165), the reductive pentose phosphate cycle (M00166, M00167), the C4-dicarboxylic acid cycle (M00170, M00171, M00172), the reductive citrate cycle (Arnon-Buchanan cycle) (M00173), the dicarboxylate-hydroxybutyrate cycle (M00374), the hydroxypropionate-hydroxybutylate cycle (M00375), the 3-Hydroxypropionate bi-cycle (M00376) and the reductive acetyl CoA pathway (M00377). A heterotroph is defined by having none of the above modules. Furthermore, we classified all genomes as chemotrophs because none of the genomes codes complete photosynthesis related modules including oxygenic photosynthesis in plants and cyanobacteria (M00611), anoxygenic photosynthesis in purple bacteria (M00612), anoxygenic photosynthesis in green nonsulfur bacteria (M00613), and anoxygenic photosynthesis in green sulfur bacteria (M00614). In addition, we defined organotroph as having any of the following modules: the glycolysis pathway (M00001 or M00002), the pentose phosphate pathway (M00004) the Entner Doudoroff pathway (M00008) or the citrate/TCA/Krebs cycle (M00009). A lithotroph is defined by coding any of the following: thiosulfate oxidation by SOX complex (M00595), nitrification (M00528), acetogen (M00618), methanogenesis (M00357, M00563, M00567). Lastly, genomes are classified as 1) aerobes when coding for the cytochrome bd ubiquinol oxidase (M00153), the cytochrome c oxidase (M00154, M00155, M00156) or the cytochrome o ubiquinol oxidase (M00417), 2) anaerobes (including fermenters) when coding for any of the following: denitrification (M00529), dissimilatory nitrate reduction (M00530), dissimilatory sulfate reduction (M00596), acetogen (M00618) and methanogenesis (M00357, M00453, M00567).

BIBLIOGRAPHY

Amos, H. M., D. J. Jacob, D. G. Streets, and E. M. Sunderland. 2013. Legacy impacts of all-time anthropogenic emissions on the global mercury cycle. *Global Biogeochemical Cycles* 27: 410–421.

Amos, H. M., D. J. Jacob, D. Kocman, H. M. Horowitz, Y. Zhang, S. Dutkiewicz, M. Horvat, E. S. Corbitt, et al. 2014. Global Biogeochemical Implications of Mercury Discharges from Rivers and Sediment Burial. *Environmental Science & Technology* 48: 9514–9522. WOS:000340701800069. doi:10.1021/es502134t.

Aramaki, T., R. Blanc-Mathieu, H. Endo, K. Ohkubo, M. Kanehisa, S. Goto, and H. Ogata. 2020. KofamKOALA: KEGG Ortholog assignment based on profile HMM and adaptive score threshold. *Bioinformatics* 36: 2251–2252. doi:10.1093/bioinformatics/btz859.

Bash, J. O. 2010. Description and initial simulation of a dynamic bidirectional air-surface exchange model for mercury in Community Multiscale Air Quality (CMAQ) model. *Journal of Geophysical Research: Atmospheres* 115. John Wiley & Sons, Ltd. doi:10.1029/2009JD012834.

Capo, E., C. Feng, A. G. Bravo, S. Bertilsson, A. L. Soerensen, J. Pinhassi, M. Buck, C. Karlsson, et al. 2022. Expression Levels of hgcAB Genes and Mercury Availability Jointly Explain Methylmercury Formation in Stratified Brackish Waters. *Environmental Science & Technology*. American Chemical Society. doi:10.1021/acs.est.2c03784.

Chaumeil, P.-A., A. J. Mussig, P. Hugenholtz, and D. H. Parks. 2020. GTDB-Tk: a toolkit to classify genomes with the Genome Taxonomy Database. *Bioinformatics* 36: 1925–1927. doi:10.1093/bioinformatics/btz848.

Dastoor, A. P., and Y. Larocque. 2004. Global circulation of atmospheric mercury: a modelling study. *Atmospheric Environment* 38: 147–161. doi:10.1016/j.atmosenv.2003.08.037.

Feinberg, A., T. Dlamini, M. Jiskra, V. Shah, and N. E. Selin. 2022. Evaluating atmospheric mercury (Hg) uptake by vegetation in a chemistry-transport model. *Environ. Sci.: Processes Impacts*. The Royal Society of Chemistry. doi:10.1039/D2EM00032F.

Gionfriddo, C., E. Capo, B. Peterson, H. Lin, D. Jones, A. G. Bravo, S. Bertilsson, J. MOREAU, et al. 2021. Hg-MATE-Db.v1.01142021. doi:10.25573/serc.13105370.v1.

Hyatt, D., G.-L. Chen, P. F. LoCascio, M. L. Land, F. W. Larimer, and L. J. Hauser. 2010. Prodigal: prokaryotic gene recognition and translation initiation site identification. *BMC Bioinformatics* 11: 119. doi:10.1186/1471-2105-11-119.

Kanehisa, M., Y. Sato, M. Kawashima, M. Furumichi, and M. Tanabe. 2016. KEGG as a reference resource for gene and protein annotation. *Nucleic Acids Research* 44: D457–D462. doi:10.1093/nar/gkv1070.

Kawai, T., T. Sakurai, and N. Suzuki. 2020. Application of a new dynamic 3-D model to investigate human impacts on the fate of mercury in the global ocean. *Environmental Modelling & Software* 124: 104599. doi:10.1016/j.envsoft.2019.104599.

Lamborg, C. H., W. F. Fitzgerald, J. O'Donnell, and T. Torgersen. 2002. A non-steady-state compartmental model of global-scale mercury biogeochemistry with interhemispheric atmospheric gradients. *Geochimica et Cosmochimica Acta* 66: 1105–1118. doi:10.1016/S0016-7037(01)00841-9.

Lei, H., X. Z. Liang, D. J. Wuebbles, and Z. Tao. 2013. Model analyses of atmospheric mercury: present air quality and effects of transpacific transport on the United States. *Atmospheric Chemistry and Physics* 13: 10807–10825. WOS:000327101900018. doi:10.5194/acp-13-10807-2013.

Mason, R. P., W. F. Fitzgerald, and F. M. M. Morel. 1994. The biogeochemical cycling of elemental mercury: Anthropogenic influences. *Geochimica et Cosmochimica Acta* 58: 3191–3198. doi:10.1016/0016-7037(94)90046-9.

Parks, D. H., M. Imelfort, C. T. Skennerton, P. Hugenholtz, and G. W. Tyson. 2015. CheckM: assessing the quality of microbial genomes recovered from isolates, single cells, and metagenomes. *Genome research* 25. Cold Spring Harbor Lab: 1043–1055.

Parks, D. H., M. Chuvochina, P.-A. Chaumeil, C. Rinke, A. J. Mussig, and P. Hugenholtz. 2020. A complete domain-to-species taxonomy for Bacteria and Archaea. *Nature Biotechnology* 38: 1079–1086. doi:10.1038/s41587-020-0501-8.

Schaefer, K., Y. Elshorbany, E. Jafarov, P. F. Schuster, R. G. Striegl, K. P. Wickland, and E. M. Sunderland. 2020. Potential impacts of mercury released from thawing permafrost. *Nature Communications* 11: 4650. doi:10.1038/s41467-020-18398-5.

Selin, N. E., D. J. Jacob, R. J. Park, R. M. Yantosca, S. S. Strode, L. Jaegle, and D. Jaffe. 2007. Chemical cycling and deposition of atmospheric mercury: Global constraints from observations. *Journal of Geophysical Research* 112: D02308.

Semeniuk, K., and A. Dastoor. 2017. Development of a global ocean mercury model with a methylation cycle: Outstanding issues. *Global Biogeochemical Cycles* 31: 400–433. doi:https://doi.org/10.1002/2016GB005452.

Shia, R.-L., C. Seigneur, P. Pai, M. Ko, and N. D. Sze. 1999. Global simulation of atmospheric mercury concentrations and deposition fluxes. *Journal of Geophysical Research: Atmospheres* 104. John Wiley & Sons, Ltd: 23747–23760. doi:10.1029/1999JD900354.

Smith-Downey, N. V., E. M. Sunderland, and D. J. Jacob. 2010. Anthropogenic impacts on global storage and emissions of mercury from terrestrial soils: Insights from a new global model. *Journal of Geophysical Research: Biogeosciences* 115. John Wiley & Sons, Ltd. doi:10.1029/2009JG001124.

Strode, S. S., L. Jaegle, N. E. Selin, D. J. Jacob, R. J. Park, R. M. Yantosca, R. P. Mason, and F. Slemr. 2007. Air-sea exchange in the global mercury cycle. *Global Biogeochemical Cycles* 1021: GB1017.

Sunderland, E. M., and R. P. Mason. 2007. Human impacts on open ocean mercury concentrations. *Global Biogeochemical Cycles* 21: GB4022.

Zhang, H., C. D. Holmes, and S. Wu. 2016. Impacts of changes in climate, land use and land cover on atmospheric mercury. *Atmospheric Environment* 141: 230–244. doi:10.1016/j.atmosenv.2016.06.056.

Zhang, P., and Y. Zhang. 2022. Earth system modeling of mercury using CESM2 – Part 1: Atmospheric model CAM6-Chem/Hg v1.0. *Geoscientific Model Development* 15: 3587–3601. doi:10.5194/gmd-15-3587-2022.

Zhang, Y., L. Jaegle, and L. Thompson. 2014. Natural biogeochemical cycle of mercury in a global three-dimensional ocean tracer model. *Global Biogeochemical Cycles* 28: 553–570. WOS:000337609600005. doi:10.1002/2014gb004814.

Zhang, Y., D. J. Jacob, S. Dutkiewicz, H. M. Amos, M. S. Long, and E. M. Sunderland. 2015. Biogeochemical drivers of the fate of riverine mercury discharged to the global and Arctic oceans. *Global Biogeochemical Cycles* in review.

Zhang, Y., H. Horowitz, J. Wang, Z. Xie, J. Kuss, and A. L. Soerensen. 2019. A Coupled Global Atmosphere-Ocean Model for Air-Sea Exchange of Mercury: Insights into Wet Deposition and Atmospheric Redox Chemistry. *ENVIRONMENTAL SCIENCE & TECHNOLOGY* 53: 5052–5061. doi:10.1021/acs.est.8b06205.

Zhang, Y., Z. Song, S. Huang, P. Zhang, Y. Peng, P. Wu, J. Gu, S. Dutkiewicz, et al. 2021a. Global health effects of future atmospheric mercury emissions. *Nature Communications* 12: 3035. doi:10.1038/s41467-021-23391-7.

Zhang, Y., S. Dutkiewicz, and E. M. Sunderland. 2021b. Impacts of climate change on methylmercury formation and bioaccumulation in the 21st century ocean. *One Earth* 4: 279–288. doi:10.1016/j.oneear.2021.01.005.

Zhou, J., D. Obrist, A. Dastoor, M. Jiskra, and A. Ryjkov. 2021. Vegetation uptake of mercury and impacts on global cycling. *Nature Reviews Earth & Environment* 2: 269–284. doi:10.1038/s43017-021-00146-y.
